# Influence of Plant Origins and Seasonal Variations on Nutritive Values, Phenolics and Antioxidant Activities of *Adenia viridiflora* Craib., an Endangered Species from Thailand

**DOI:** 10.3390/foods10112799

**Published:** 2021-11-14

**Authors:** Werawat Wannasaksri, Piya Temviriyanukul, Amornrat Aursalung, Yuraporn Sahasakul, Sirinapa Thangsiri, Woorawee Inthachat, Nattira On-Nom, Chaowanee Chupeerach, Kanchana Pruesapan, Somsri Charoenkiatkul, Uthaiwan Suttisansanee

**Affiliations:** 1 Institute of Nutrition, Mahidol University, Salaya, Phuttamonthon, Nakhon Pathom 73170, Thailand; nit.frank@gmail.com (W.W.); piya.tem@mahidol.ac.th (P.T.); amornrat.aur@mahidol.ac.th (A.A.); yuraporn.sah@mahidol.ac.th (Y.S.); sirinapa.thang@outlook.com (S.T.); woorawee.int@mahidol.ac.th (W.I.); nattira.onn@mahidol.ac.th (N.O.-N.); chaowanee.chu@mahidol.ac.th (C.C.); somsri.chr@mahidol.ac.th (S.C.); 2 Plant Varieties Protection Division, Department of Agriculture, Ministry of Agriculture and Cooperatives, Bangkok 10900, Thailand; kpruesapan@gmail.com

**Keywords:** indigenous plant, leaves, origins, harvesting times, nutrients, secondary metabolites, antioxidants

## Abstract

*Adenia viridiflora* Craib. is an indigenous plant found in Thailand, Cambodia and Vietnam that has become threatened owing to lack of knowledge about its agricultural management. This plant is now rare in the wild and was registered in the Plant Genetic Conservation Project under the initiation of Her Royal Highness Princess Maha Chakri Sirindhorn (RSPG) to promote sustainable conservation and optimally beneficial utilization. *A. viridiflora* has a long history of utilization as a nutrient-rich source with medicinal properties but scientific evidence of the veracity of these claims is limited. Here, the nutritional compositions, phenolic contents and antioxidant activities of different plant parts (young shoots and old leaves) of *A. viridiflora* were investigated using plants collected from four areas of Thailand as Kamphaeng Phet (KP), Muang Nakhon Ratchasima (MN), Pakchong Nakhon Ratchasima (PN) and Uthai Thani (UT) at different harvesting periods (March-April, May–June and July–August). Results indicated that young shoots provided higher energy, protein, fat, dietary fiber, phosphorus, sodium, and zinc than old leaves. By contrast, nutrients such as total sugar, vitamin C, carotenoids, potassium, calcium, magnesium, and iron contents were higher in old leaves that also exhibited higher phenolic contents and most antioxidant activities than young shoots. Generally, most nutrients, phenolic contents, and antioxidant activities exhibited no clear trend among different plant origins. The harvesting period of July–August provided a suitable climate for biosynthesis of most nutrients, while high phenolics were mainly found in samples harvested in March–April. No clear trend was observed in the prevalence of antioxidant activities that varied according to assay techniques.

## 1. Introduction

*Adenia viridiflora* Craib. (or Pak E-noon in Thai) is a member of the *Passifloraceae* family. This species is indigenous to Thailand, Cambodia, and Vietnam and grows in the wild by climbing on other trees to a height of 20 m. In Thailand, the plant is found in mixed deciduous and deciduous dipterocarp forest in the northeast of the country (especially in Uthai Thani, Nakhon Sawan, Nakhon Ratchasima, Kamphaeng Phet, Lop Buri Provinces, and nearby areas). *A. viridiflora* grows as a smooth, dark green round vine that turns rough and light brown with age. The dark green to violet-red shoots have tendrils that attach to other trees. This monocot plant has heart-shaped leaves, and its flowers bloom from late winter to the early rainy season. Young fruit is green with a diameter of 4–5 cm and turns yellow-orange to violet when reaching maturity. The flowers, young fruits, shoots and leaves form the edible parts of the plant and can be harvested from March to August. The plant assumes a resting state during periods of drought (September–February). The edible parts are blanched or fermented with water from washing rice and consumed with spicy sauce as a vegetable side dish. Consumption of this endemic species is limited to indigenous populations, and it is rarely found in local markets.

*A. viridiflora* can be used as a traditional herb to treat urinary tract infection, fever, giddiness, fainting, and diarrhea [1]. Our previous study, as the only report citing scientific-based evidence of potential health properties [2], determined that an aqueous extract of *A. viridiflora* inhibited key enzymes related to obesity (lipase), diabetes (α-glucosidase and dipeptidyl peptidase-IV), hypertension (angiotensin-converting enzyme), and Alzheimer’s disease (cholinesterases and β-secretase), suggesting high potential for further development as a functional food. In the wild, *A. viridiflora* is an endangered species due to unlimited local consumption and poorly known reproduction and growth conditions. The plant was registered in the Plant Genetic Conservation Project under the initiation of Her Royal Highness Princess Maha Chakri Sirindhorn (RSPG) in 2010.

To improve information on plant cultivation, nutritional aspects, and bioactivities, this research investigated the nutritional compositions, phenolic contents, and antioxidant activities of edible plant parts (young shoots and old leaves) of *A. viridiflora* to promote its consumption and potential development as a food product. Different origins (Kamphaeng Phet (KP), Muang Nakhon Ratchasima (MN), Pakchong Nakhon Ratchasima (PN), and Uthai Thani (UT)), and harvesting periods (March–April, May–June and July–August) of *A. viridiflora* were also investigated to expand knowledge on agricultural management and potential plant genomic development. This is the first report detailing the nutritional compositions of different plant parts of *A. viridiflora* collected from diverse sources at various harvesting times. Knowledge gained from this research can be used to promote *A. viridiflora* as a healthy vegetable for consumption and initiate agricultural stimulation. This plant shows great promise for application in future functional food development that can lead to sustainable conservation and utilization as the ultimate goal of the RSPG.

## 2. Materials and Methods

### 2.1. Sample Preparation and Extraction

*Adenia viridiflora* Craib. was collected from Kamphaeng Phet (KP), Muang Nakhon Ratchasima (MN), Pakchong Nakhon Ratchasima (PN), and Uthai Thani (UT), Thailand. The plants were then cultivated at Khlong Phai sub-district, Sikhio district, Nakhon Ratchasima province, Thailand (14°86′18.7″ N and 101°56′83.3″ E), which is the conservative plant areas for experimental botanical purposes of the Plant Genetic Conservation Project under the royal initiation of Her Royal Highness Princess Maha Chakri Sirindhorn (RSPG). Young shoots (length 30 cm from the top) and old leaves (length 30–50 cm from the top) were harvested during March–April, May–June and July–August, 2018. Statistics of rainfall at the meteorology station (M.38C, Sikhio district, Nakhon Ratchasima province, Thailand) were received from the Lower Northeastern Region Hydrological lrrigation Center, Bureau of Water Management and Hydrology, Royal Irrigation Department, Thailand (http://hydro-4.rid.go.th (accessed on 16 March 2021)) and was reported in Table 1. The physical appearances of all samples were shown in Supplementary Tables S1 and S2. All plant samples were identified and authenticated by Asst. Prof. Dr. Renu Khumlert and Dr. Aschan Sukthumrong from Institute of Agricultural Technology, Suranaree University of Technology, Nakhon Ratchasima, Thailand. The plants were deposited at the Bangkok Herbarium (BK), Bangkok, Thailand, and assigned voucher specimens as BK No. 071408 (KP), BK No. 071410 (MN), BK No. 071411 (PN), and BK No. 071409 (UT).

The samples were cleaned with deionized water, air-dried for 2–3 h and freeze-dried for 3 days using a Heto PowerDry PL9000 freeze dryer (Heto Lab Equipment, Allerod, Denmark). The dry samples were then ground using a Philips 600 W grinder (Philips Electronic Co., Ltd., Jakarta, Indonesia) into fine powder and were kept at –20 °C until analysis. 

The color analysis of fresh and dried samples was performed using a ColorFlex EZ spectrophotometer (Hunter Associates Laboratory, Reston, VA, USA), and the results were expressed as CIELAB units, in which L * represents dark (0) to white (100), a * represents green (−) to red (+) colors, and b * represents blue (−) to yellow (+) colors, as shown in Supplementary Table S3. The determination of moisture contents was performed using a Halogen HE53 moisture analyzer (Mettler Toledo AG, Greifensee, Switzerland), and the results were expressed as a percentage of moisture content, as shown in Supplementary Table S4. 

While fresh samples were used for analysis of nutritional compositions, dry samples were extracted using distilled water as previously reported [2] and used for determination of total phenolic contents, total flavonoid contents, and antioxidant capacities. Briefly, the mixture of powdery sample in distilled water (0.5 g dry weight/10 mL) was incubated at 50 °C for 2 h using a WNE45 water bath shaker (Memmert GmBh, Eagle, WI, USA). The supernatant was collected from a Hettich^®^ ROTINA 38R centrifugation (Andreas Hettich GmbH, Tuttlingen, Germany) of the mixture at 3800× *g* for 15 min, followed by a filtration through a 0.45 µM polyethersulfone membrane (PTFE) syringe filter.

### 2.2. Determination of Nutritive Values

The nutritive values were analyzed using the standard protocols of the Association of Official Analytical Chemists (AOAC) [3] as previously described [4] and performed at the Institute of Nutrition, Mahidol University with ISO/IEC 17025:2005. Nutritional values including moisture content, energy, fat, protein, carbohydrate, total sugar, fructose, glucose, sucrose, total dietary fiber, soluble dietary fiber, insoluble dietary fiber, ash, vitamin C, and minerals (Ca, K, Mg, Na, Fe, and Zn) were reported as per 100 g fresh weight, as shown in Supplementary Tables S5–S8. To accurately determine the effect of the seasonal variation, plant origins, and plant parts, nutritional contents were calculated and reported as per 100 g dry weight.

#### 2.2.1. Moisture Content

Moisture content was evaluated by incubating a fresh sample in a Memmert UNE 500 hot-air oven (Eagle, WI, USA) at 100 °C until the sample weight was unchanged (AOAC 930.04, 934.01).

#### 2.2.2. Protein

Protein was analyzed according to the Kjeldahl method with Büchi K-435 digestion and Büchi B-324 distillation units (BÜCHI Corporation, New Castle, DE, USA). Protein content was calculated using a conversion factor of 6.25 (AOAC 991.20).

#### 2.2.3. Fat

Fat was determined using acidic digestion and petroleum ether extraction in a Tecator Soxtec System HT 1043 (Foss Tecator, Hoganas, Sweden) (AOAC 948.15, 945.16).

#### 2.2.4. Dietary Fiber

Total dietary fiber, soluble dietary fiber (SDF), and insoluble dietary fiber (IDF) were investigated using the enzymatic (α-amylase, protease, and amyloglucosidase) gravimetric method (AOAC 993.19 for SDF and AOAC 991.42 for IDF). Total dietary fiber was calculated from the sum of SDF and IDF.

#### 2.2.5. Sugars

Monosaccharides and disaccharides (fructose, glucose, and sucrose) were identified and quantified according to the previous report [5] with some modifications as follows. An ultra-fast liquid chromatography (UFLC) equipped with an Alltech *800 evaporative light scattering* detector (ELSD) (BÜCHI Corporation, New Castle, DE, USA), a LC-20AD pump (Shimadzu Corporation, Kyoto, Japan) and a 5 µm, 250 × 4.6 mm Shodex Asahi Pak NH2P-50 4E column (Shodex Group, Kanagawa, Japan) was set up with an isocratic solvent of 76% (*v*/*v*) acetonitrile and a flow rate of 1.0 mL/min. Total sugar content was calculated from the sum of these monosaccharides and disaccharides.

#### 2.2.6. Ash

Ash was determined by incineration at 550 °C in a Carbolite CWF 1100 muffle furnace (Carbolite Gero Ltd., Hope, UK) (AOAC 930.30, 945.46).

#### 2.2.7. Carbohydrate

Carbohydrate was calculated from moisture, protein, fat, and ash contents using Equation (1):

Total carbohydrate (g) = 100 − moisture (g) − protein (g) − total fat (g) − ash (g)(1)

#### 2.2.8. Energy

The energy was calculated from carbohydrate, protein, and fat contents using Equation (2):

Energy (kcal) = (total carbohydrate × 4) + (protein × 4) + (total fat × 9)(2)

#### 2.2.9. Vitamin C

Vitamin C was determined as previously reported [6] using high-performance liquid chromatography (HPLC) equipped with an UV-975 UV/Vis detector (JASCO International Co., Ltd., Tokyo, Japan), a Waters 515 pump (Waters Corporation, Milford, MA, USA), and a 5 μm, 250 × 4.6 mm Zorbax original ODS column (Agilent Technologies, Santa Clara, CA, USA). The HPLC system employed an isocratic solvent system of 0.5% (*v*/*v*) KH_2_PO_4_ (adjusted to pH 2.5 with H_3_PO_4_) with a 0.8 mL/min flow rate. Vitamin C was visualized at 254 nm.

#### 2.2.10. Carotenoids

Carotenoids were evaluated as previously reported [7] using an Agilent 1100 HPLC system equipped with a photodiode array detector (Agilent Technologies) and a 5 µm, 150 × 4.6 mm Vydac 201TP-C18 column (Alltech Associates, Inc., Columbia, MD, USA). The HPLC system employed the gradient mobile phases of absolute methyl tert-butyl ether (solvent A) and methanol containing 2% (*v*/*v*) ammonium acetate (solvent B) with a constant flow rate of 0.6 mL/min and a detection at 450 nm. The standards including α-carotene (>95.0% HPLC), β-carotene (>95.0% HPLC), β-cryptoxanthin (>97.0% TLC), lutein (>96.0% HPLC), lycopene (>98.0% HPLC), and zeaxanthin (>95.0% HPLC) were received from Sigma-Aldrich (St. Louis, MO, USA). 

#### 2.2.11. Minerals

Ash was also used to evaluate Ca, Na, and K contents using a Thermo S series flame atomic absorption spectrophotometer (Thermo Electron Corporation, Cambridge, UK) (AOAC 985.35), while Mg, Fe, and Zn contents were analyzed using an Optima 4200 DV inductively coupled plasma optical emission spectroscopy (PerkinElmer^®^, Waltham, MA, USA) (AOAC 984.27).

### 2.3. Determination of Total Phenolic Contents 

Total phenolic contents (TPCs) were performed according to a well-established protocol as previously described [8] using a Folin–Ciocalteu reagent and gallic acid standard (0–200 µg/mL). The TPCs were monitored at 765 nm using a Synergy^TM^ HT 96–well UV–visible microplate reader (BioTek Instruments, Inc., Winooski, VT, USA) with a Gen 5 data analysis software. 

Total flavonoid contents (TFCs) were performed according to a well-established protocol as previously described [9] using sodium nitrite and aluminum chloride hexahydrate reagents, while quercetin was used as a standard (0–100 µg/mL). The TFCs were monitored at 510 nm using the 96-well UV–visible microplate reader.

### 2.4. Determination of Antioxidant Activities

The antioxidant activities were determined using 2,2-diphenyl-1-picrylhydrazyl (DPPH) radical scavenging, ferric ion reducing antioxidant power (FRAP), and oxygen radical absorbance capacity (ORAC) assays as previously described [10]. The antioxidant activity determined by a DPPH radical scavenging assay was evaluated using a DPPH reagent, while an FRAP reagent was used in an FRAP assay and a fluorescein reagent in ORAC assay. Trolox, a water soluble vitamin E analogue, was used as a standard. The end-point detections at 520 and 595 nm were employed for DPPH radical scavenging and FRAP assays, respectively, while the kinetic detection at an excitation wavelength of 485 nm and emission wavelength of 528 nm was for an ORAC assay using the 96-well UV–visible microplate reader.

### 2.5. Statistical Analysis

The results were expressed as mean ± standard deviation (SD) of triplicate experiments (*n* = 3). Statistical analysis at *p* < 0.05 was performed using one-way analysis of variance (ANOVA), followed by Duncan’s multiple comparison test (more than two data), or an unpaired *t*-test (two data). 

Principal component analysis (PCA) and hierarchical cluster analysis (HCA) of nutritive components, TPCs, TFCs and antioxidant activities were analyzed using XLSTAT^®^ (Addinsoft Inc., New York, NY, USA) by Panyaporn Kanoongon. 

## 3. Results

### 3.1. Nutritive Values

Nutritive values including energy, moisture, protein, fat, total carbohydrate, total dietary fiber, SDF, IDF, total sugar, fructose, glucose, sucrose, ash, vitamin C, and minerals (Ca, P, Na, K, Mg, Fe, and Zn) of young shoots and old leaves with different origins collected at diverse harvesting times were determined by the AOAC method. Results are reported per 100 g fresh weight (FW) in Supplementary Tables S5–S8, with nutritional contents per 100 g dry weight (DW) shown in Table 2, Table 3, Table 4 and Table 5. These values were used to investigate the effect of plant parts and origins without the variable of moisture content.

Fresh young shoots of *Adenia viridiflora* Craib. gave moisture contents of 83.69–86.87% (Supplementary Table S4), with UT exhibiting lower moisture contents than the other collection areas. Fresh samples collected in May–June had slightly lower moisture contents than the other harvesting periods. Fresh old leaves exhibited moisture contents of 78.00–83.08%, with PN and UT showing higher moisture contents than MN and KP. Similar to young shoots, most old leaves collected in May–June exhibited lower moisture contents than the other two harvesting periods. Moisture contents decreased 10–16 times after the drying process, with moisture contents of dried samples ranging from 5.18–7.81%.

Results indicated that young shoots of *A. viridiflora* provided 371.45–387.69 kcal of energy, while old leaves gave 373.22–386.97 kcal (Table 2). Comparing plant parts, most young shoots exhibited slightly higher energy than old leaves. Young shoots of UT collected in March–April and May–June provided the lowest energy, while young shoots of KP give the lowest energy among the samples collected in July–August. In addition, the lowest energy was also observed in old leaves from MN collected in all harvesting periods. However, the effect of seasonal variation on energy was unclear. For example, young shoots of KP collected during May–June provided the higher energy, while in the same harvesting period, young shoots of MN gave the lowest energy. Nevertheless, the energy in young shoots and old leaves of all plant origin collected during different harvesting periods was varied in a close range (within 4%).

Protein contents in young shoots of *A. viridiflora* were similar, ranging 18.15–21.94 g, while old leaves exhibited protein ranging 16.53–22.68 g (Table 2). Most young shoots had slightly higher protein contents than old leaves, especially the one collected during May–June. Young shoots and old leaves from PN gave higher protein contents than other origins collected in the same harvesting period, except young shoots collected during March–April and old leaves collected during July–August. Interestingly, most samples collected in March–April potentially provided higher protein contents than those collected during other harvesting periods.

Young shoots exhibited fat contents ranging 0.62–4.10 g, while old leaves gave 1.66–3.28 g (Table 2). Most young shoots had higher fat contents than old leaves (up to 1.4 times); however, old leaves of PN and UT collected in March–April exhibited significantly higher fat contents than their corresponding young shoots (5.0 and 4.2 times, respectively). Young shoots of KP tended to provide higher fat content than other origin, while similar fat contents were observed in old leaves regardless of plant origins. The effect of seasonal variation on fat content in young shoots was unclear even though young shoots from PN and UT collected during July–August tended to provide higher contents than those collected during other harvesting periods. However, fat contents in old leaves seemed to be unaffected by season variation.

Similar carbohydrate contents in young shoots ranging 67.21–74.57 g and old leaves ranging 67.12–73.53 g were recorded (Table 2). However, the effect of plant origin and seasonal variation on carbohydrate contents was unclear. All samples exhibited similar carbohydrate contents in all harvesting periods regardless of origin.

Ash (6.67–9.12 g in young shoots and 7.11–9.17 g in old leaves) was used to calculate the amount of minerals (Table 2). Ash contents in young shoots were mostly lower than their corresponding old leaves. Young shoots of UT and KP likely exhibited higher ash contents than other origins, while similar results were observed with old leaves of MN and KP. Interestingly, the samples collected in May–July exhibited higher ash contents than those collected during other harvesting periods.

As one type of carbohydrate, total dietary fiber contents were evaluated as a sum of soluble and insoluble dietary fibers (Table 3). Total dietary fiber contents in young shoots ranged from 32.89–65.26 g, while old leaves contained 28.37–51.46 g. Young shoots tended to exhibit higher fiber contents than old leaves (up to 2.13 times). Young shoots of UT had higher fiber contents than other origins collected at the same harvesting period, while no clear trend was observed in old leaves. Interestingly, samples collected in July–August tended to provide higher fiber contents than those collected at other harvesting times. All samples exhibited lower soluble dietary fiber (SDF) than insoluble dietary fiber (IDF). Young shoots and old leaves contained wide-ranging SDF (3.43–20.47 and 4.32–18.96 g, respectively), while old leaves exhibited potentially higher SDF contents than young shoots. By contrast, young shoots contained higher IDF contents (24.70–47.77 g) than old leaves (23.46–38.60 g). Young shoots of UT exhibited higher SDF and IDF than other origins harvested at the same time, while old leaves of PN and KP exhibited higher SDF and IDF contents, respectively, than the other origins. Samples collected in May–June contained higher SDF than other harvesting periods, while July–August provided high IDF contents.

Total sugar contents were evaluated as a sum of fructose, glucose, and sucrose contents (Table 3). Total sugar contents of young shoots ranged from 8.45–16.16 g, with old leaves 11.83–18.22 g. Old leaves exhibited higher sugar contents than young shoots. Young shoots of UT had higher sugar contents than other origins collected during the same harvesting period, while no clear trend was observed in old leaves. Interestingly, samples collected in July–August exhibited higher sugar contents than those collected at other times. Types of sugar detected in both young shoots and old leaves included fructose, glucose, and sucrose. Glucose, as the main component of sugar, accounted for 52–92% of total sugar in young shoots and 55–67% of total sugar in old leaves. The second most abundant sugar, fructose, accounted for 8–48% of total sugar in young shoots and 28–39% of total sugar in old leaves, while sucrose as the least abundant sugar, accounted for up to 13% of total sugar in young shoots and up to 17% in old leaves. Similarly, old leaves exhibited higher glucose, fructose, and sucrose contents than young shoots. However, no clear trend on the effect of plant origins on fructose, glucose, and sucrose contents was observed. Samples collected in July–August provided higher fructose, glucose, and sucrose contents than at other times, especially sucrose, which was only detected in young shoots collected in July–August.

*A. viridiflora* provided high vitamin C contents that ranged from 573.93–1696.46 mg in young shoots and 862.64–2240.65 mg in old leaves (Table 4). Old leaves provided 1.1–1.9 times higher vitamin C contents than young shoots, while vitamin C contents in both plant parts of UT were higher than from other origins. Samples collected in July–August tended to exhibit higher contents than those collected at other harvesting times.

Among the carotenoid standards (lutein, zeaxanthin, β-cryptoxanthin, α-carotene, β-carotene and lycopene), high-performance liquid chromatographic (HPLC) results indicated that only lutein and β-carotene were detected in *A. viridiflora* (Table 4). Young shoots exhibited lutein as the most abundant carotenoid ranging 26.47–65.20 mg, while 50.26–93.49 mg were found in old leaves. Lower contents of β-carotene were also detected in young shoots (12.08–27.24 mg) and old leaves (31.52–49.84 mg). Interestingly, old leaves exhibited significantly higher lutein (1.4–2.6 times) and β-carotene (1.7–3.6 times) contents than young shoots, while KP provided higher carotenoid contents than the other origins. Samples collected in March–April tended to exhibit higher contents than those collected at other harvesting periods.

Macrominerals detected in *A. viridiflora* included potassium, calcium, phosphorus, magnesium, and sodium (Table 5). As the most abundant macromineral, potassium contents in young shoots ranged 1740.09–2549.33 mg (accounting for 51–63% of total macrominerals), while old leaves contained 1357.16–2144.11 mg (accounting for 34–50% of total macrominerals). Higher potassium content (1.1–1.5 times) was found in old leaves than in young shoots, while UT exhibited higher contents than the other origins. Higher potassium content was found in samples collected in July–August than at other harvesting periods. Young shoots contained calcium ranging 397.97–907.11 mg and old leaves 968.40–1645.10 mg (accounting for 12–24% and 23–40% of total macrominerals, respectively). Similar to potassium, old leaves exhibited 1.6–2.5 times higher calcium than young shoots, while MN provided higher contents than the other origins. May–June also gave higher calcium contents than the other harvesting periods. For phosphorus, young shoots contained 467.56–601.59 mg, while old leaves contained 401.93–477.53 mg (accounting for 12–18% and 9–14% of total macrominerals, respectively). Unlike potassium and calcium, young shoots contained slightly higher phosphorus contents than old leaves. Similar phosphorus contents were detected in young shoots regardless of plant origins, while old leaves of UT tended to exhibit higher phosphorus contents than the other origins for the same plant part. Interestingly, phosphorus contents in young shoots were unaffected by harvesting periods, while the effect was unclear in old leaves. Higher magnesium contents were found in old leaves (305.46–951.84 mg, accounting for 6–11% of total macrominerals) than in young shoots (217.54–473.54 mg, accounting for 8–20% of total macrominerals), with MN providing higher content than the other origins. Higher magnesium contents were observed in samples collected in July–August than those collected during other harvesting periods. As the least abundant among macrominerals (accounting for 1–4% of the total), higher sodium contents were found in most young shoots (51.74–152.02 mg) than in old leaves (30.65–131.97 mg). Young shoots exhibited similar sodium contents in all harvesting periods regardless of origin, while old leaves of PN tended to exhibit higher contents than other origins when considering the same plant part. Overall sodium contents were unaffected by seasonal variation.

Microminerals including iron and zinc (accounting for 53–66% and 27–42% of total microminerals, respectively) were also investigated (Table 5). Young shoots contained iron ranging from 4.27–7.08 mg, while old leaves exhibited 4.90–8.57 mg. Old leaves provided slightly higher iron contents than young shoots. No clear trends were observed in iron content for plant origins and harvesting seasons. Zinc contents in young shoots ranged from 2.25–3.89 mg and in old leaves 2.59–3.90 mg. Young shoots had higher zinc contents than old leaves, while KP tended to exhibit higher zinc content than the other origins. However, no clear trend in zinc content was observed for harvesting periods.

### 3.2. Total Phenolic Contents and Total Flavonoid Contents

Total phenolic contents (TPCs) of all samples ranged from 18.94–28.70 mg gallic acid equivalent (GAE)/g DW (Table 6). Young shoots exhibited TPCs ranging from 18.94–26.35 mg GAE/g DW, while old leaves 23.56–28.70 mg GAE/g DW, suggesting that most old leaves from the same plant origins collected during the same harvesting period exhibited higher TPCs than their corresponding young shoots. Young shoots from MN had higher TPCs than other origins collected in March–April and May–June, while old leaves from UT collected in May–June and July–August had higher TPCs than those from other origins collected during the same harvesting periods. Interestingly, young shoots from KP collected in July–August and old leaves from KP collected in March–April exhibited higher TPCs than other origins collected in the same harvesting periods. For seasonal variation, most samples harvested in March–April exhibited higher TPCs than at the other harvesting periods.

Total flavonoid contents (TFCs) of all samples ranged from 5.37–14.08 mg quercetin equivalent (QE)/g DW (Table 6). Old leaves exhibited TFCs ranging from 5.96–14.08 mg QE/g DW, while young shoots gave 5.37–10.91 mg QE/g DW, suggesting that most old leaves from the same plant origins collected during the same harvesting period had higher TFCs than their corresponding young shoots. Young shoots from PN exhibited higher TFCs than from other plant origins collected in March–April and May–June, while young shoots from KP had higher TFCs than from other origins collected during July–August. Old leaves from MN and PN had higher TFCs than from other origins collected in all harvesting periods, while most samples collected in July–August exhibited higher TFCs than at the other harvesting periods.

### 3.3. Antioxidant Activities

Three methods were employed to measure antioxidant activities as 2,2-diphenyl-1-picrylhydrazyl (DPPH) radical scavenging, ferric reducing antioxidant power (FRAP) and oxygen radical antioxidant capacity (ORAC) assays (Table 6). The first two followed a single electron transfer (SET) mechanism, while the third involved hydrogen atom transfer (HAT) mechanism. 

Results for DPPH radical scavenging activities ranged from 0.78–1.44 µmol Trolox equivalent (TE)/100 g DW. Young shoots exhibited DPPH radical scavenging activities ranging from 0.78–1.37 µmol TE/100 g DW, while old leaves gave 0.96–1.44 µmol TE/100 g DW, suggesting that most old leaves from the same plant origin collected in the same harvesting period exhibited higher DPPH radical scavenging activities than their corresponding young shoots. Most young shoots harvested in March–April exhibited higher radical scavenging activities than their corresponding old leaves. Young shoots from KP exhibited higher radical scavenging activities than other origins in most cases, while the corresponding old leaves from KP exhibited the least radical scavenging activity compared to the other plant origins. Young shoots collected in March–April and old leaves collected in July–August exhibited higher radical scavenging activities than the same plant parts collected during other harvesting periods. 

Reducing activities by FRAP assay of all samples ranged from 14.36–42.90 µmol TE/g DW. Old leaves exhibited higher reducing activities (27.08–42.90 µmol TE/g DW) than young shoots (14.36–29.24 µmol TE/g DW). Young shoots of MN and PN exhibited higher reducing activities than those from other origins collected in March–April and May–June, while young shoots of KP collected in July–August exhibited highest reducing activities. Likewise, old leaves of PN exhibited higher reducing activities than those from other origins collected in March–April and July–August, while old leaves from UT harvested in May–June exhibited higher reducing activities than from other origins harvested at the same time. The effect of harvesting time on reducing activities varied in young shoots, with no trend observed. However, old leaves harvested in May–June exhibited higher reducing activities than at other harvesting times.

All plant samples exhibited ORAC activities ranging from 592.08–1438.85 µmol TE/g DW, with young shoots recording 649.06–1438.85 µmol TE/g DW and old leaves 592.08–1403.53 µmol TE/g DW. In most samples, young shoots gave higher ORAC activities than old leaves, while PN exhibited higher ORAC activities than other origins collected at the same harvesting period (with the exception of old leaves collected in July–August). Samples collected in March–April yielded higher ORAC activities than at other harvesting times.

### 3.4. Correlation by Principal Component Analysis (PCA) and Hierarchical Cluster Analysis (HCA)

**Figure 1:**
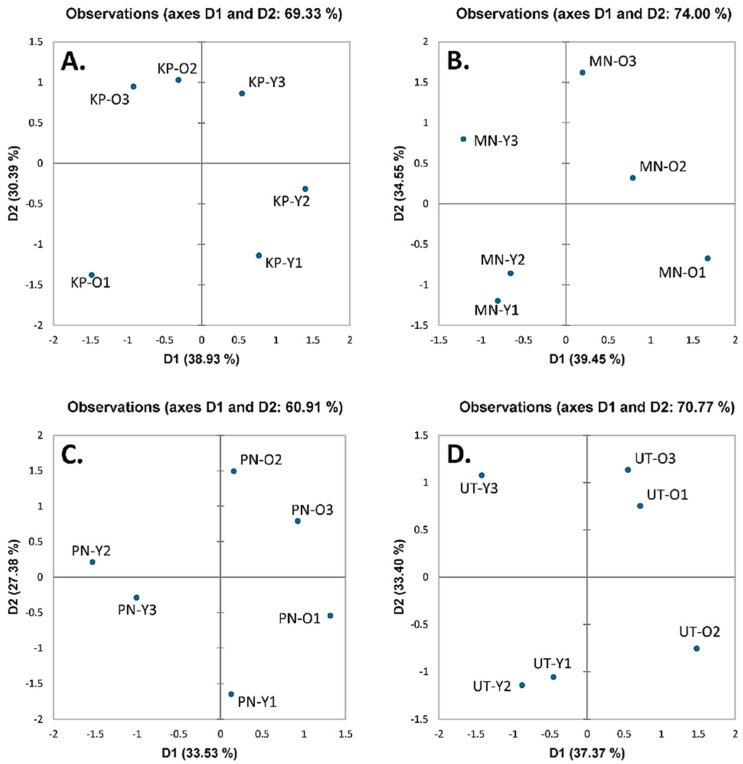
Observational plot of Principal Component Analysis (PCA) derived from mean of nutritive values, total phenolic contents, total flavonoid contents, and antioxidant activities in young shoots (Y) and old leaves (O) of *Adenia viridiflora* Craib. from different origins including (**A**) Kamphaeng Phet (KP); (**B**) Muang Nakhon Ratchasima (MN); (**C**) Pakchong Nakhon Ratchasima (PN); and (**D**) Uthai Thani (UT) collected in different harvesting periods including March–April (1), May–June (2), and July–August (3).

Abundant nutritive values of *A. viridiflora* were recorded from different origins and seasons, with no clear conclusions drawn. Thus, principal component analysis (PCA) was employed to analyze data correlation using mean nutritive values, TPCs, TFCs, and antioxidant activities (Table 2, Table 3, Table 4, Table 5 and Table 6). Data from PCA indicated that all samples (KP, MN, PN, and UT) were graphically scattered, indicating the absence of relationships (Figure 1), while both plant parts and seasonal variation influenced nutritional quality, TPCs, TFCs, and antioxidant properties. 

**Figure 2:**
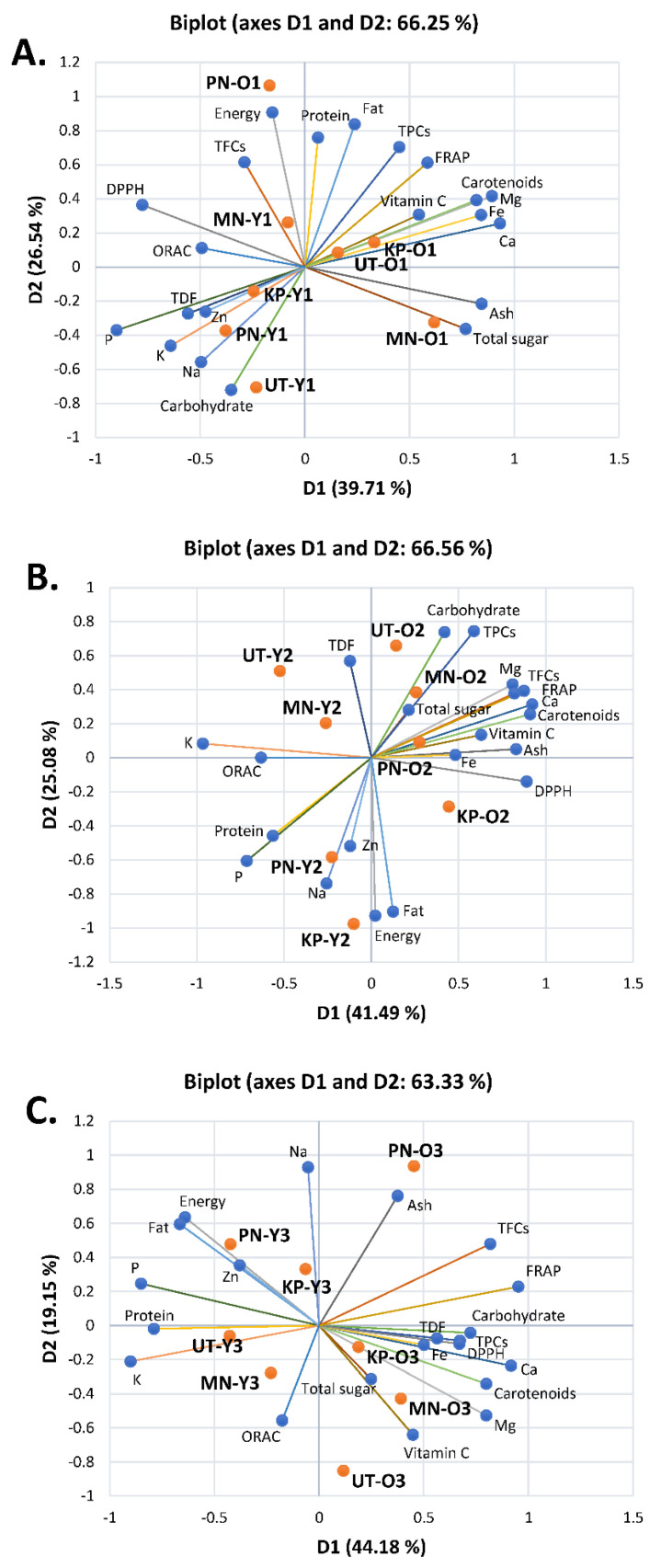
Biplot of Principal Component Analysis (PCA) derived from mean of nutritive values, total phenolic contents, total flavonoid contents, and antioxidant activities in young shoots (Y) and old leaves (O) of *Adenia viridiflora* Craib. from different origins including Kamphaeng Phet (KP), Muang Nakhon Ratchasima (MN), Pakchong Nakhon Ratchasima (PN), and Uthai Thani (UT) collected in different harvesting periods including (**A**) March–April (1); (**B**) May–June (2); and (**C**) July–August (3).

To further observe the relationships between the observations (samples) and variables (their nutritional compositions, TPCs, TFCs, and antioxidant activities) within the same season, biplots were created as shown in Figure 2. The first two axes were assigned as PC1 and PC2 covering 66.25%, 66.56%, and 63.33% of all variables from March–April (Figure 2A), May–June (Figure 2B) and July–August (Figure 2C), respectively. Interestingly, the biplots showed that, in all seasons, old leaves of *A. viridiflora* were good sources of nutritive values and antioxidant properties as the old leaves were projected closer to most variables than young shoots. Old leaves of MN, KP and UT collected in March–April were rich in some nutrients such as carotenoids and sugar and also contained Fe and Ca (Figure 2A), while old leaves of PN projected almost perpendicularly to the others, suggesting an uncorrelated relationship between old leaves of PN and other origins. Intriguingly, old leaves from all origins collected in May–June were rich in nutrients such as carbohydrates, most minerals and TPCs and also showed antioxidant activities (Figure 2B), while old leaves of KP, MN, and UT collected in July–August were rich in nutrients including fiber, vitamin C, some minerals such as Mg, and also had antioxidant properties as they were located close to most variables (Figure 2C). The PCA data indicated that seasonal variations influenced nutritional compositions, TPCs, TFCs, and antioxidant properties of *A. viridiflora* from the four origins. KP was highly impacted, while MN was highly correlated with these variables, showing minor effects from seasonal variations. 

**Figure 3:**
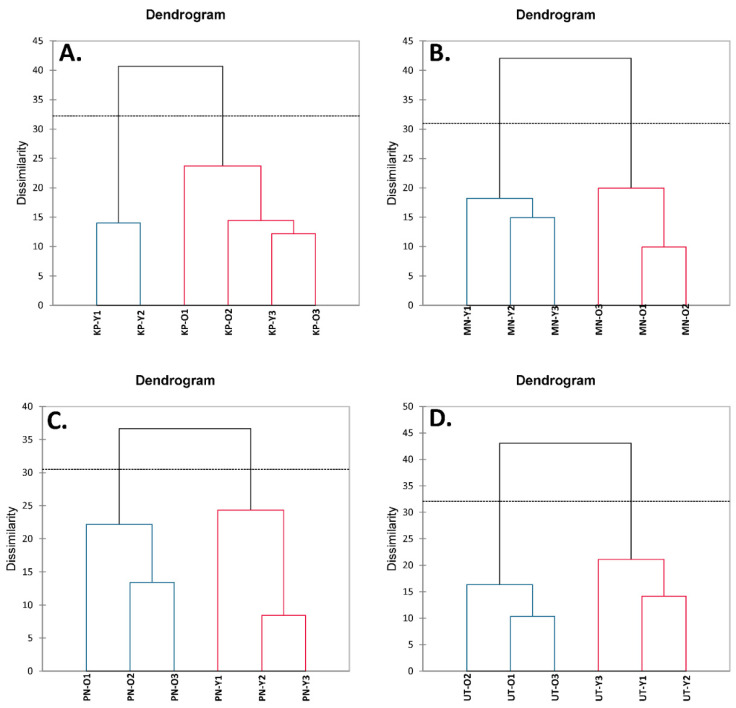
The dendrogram of agglomerative hierarchical cluster analysis (AHC) derived from mean of nutritive values, total phenolic contents, total flavonoid contents, and antioxidant activities in young shoots (Y) and old leaves (O) of *Adenia viridiflora* Craib. from different origins including (**A**) Kamphaeng Phet (KP); (**B**) Muang Nakhon Ratchasima (MN); (**C**) Pakchong Nakhon Ratchasima (PN); and (**D**) Uthai Thani (UT) collected in different harvesting periods including March–April (1), May–June (2), and July–August (3).

Agglomerative hierarchical clustering (AHC) was used to analyze the clustering of the tested *A. viridiflora* samples by clustering similar objects together (Figure 3). Results indicated that young shoots of MN, PN, and UT were clustered together with their old leaves (Figure 3B–D), suggesting a clear cluster between young and old stages of these three cultivars. However, young shoots and old leaves of KP were not well-clustered (Figure 3A). The PCA and AHC data implied that seasonal variations affected MN, PN, and UT in the same pattern but were incomparable with KP. To control sample quality, MN was the recommended cultivar for future farming since this origin was less affected by seasonal variations.

## 4. Discussion

*Adenia viridiflora* Craib. found mostly in forested areas of Northern Thailand is an endangered species. Agricultural knowledge and marketing availability on this plant are lacking, with limited information on its nutritional contents, contained bioactive compounds and health benefits. Previous reports on nutritional values and medicinal applications of other related species [11,12,13] suggested that *Adenia* might be a rich source of nutrients with advantageous health benefits. This is the first report detailing the nutritional contents of young shoots and old leaves of *A. viridiflora* collected from different geographical areas at diverse harvesting times. Results indicated that plant parts and seasonal variation had greater impact on nutritional compositions, phenolic contents, and antioxidant activities than plant origins. We found that (i) young shoots provided higher energy and most nutrients (protein, fat, and dietary fiber) than old leaves, while the latter exhibited higher sugar, vitamin C, carotenoids, and most minerals than the former, (ii) old leaves exhibited higher phenolic contents with greater antioxidant activities than young shoots, and (iii) samples collected in July–August possessed high nutrients, while those collected in March–April exhibited high phenolics, and (iv) the impact of plant origins on nutritional compositions, phenolic contents, and antioxidant activities remained unclear.

A previous study reported that *A. cissampeloides* exhibited energy of 408 kcal with 25% protein, 25% fat, 22% carbohydrate, and 13% fiber [11]. These data concurred with the nutritive values recorded for *A. viridiflora* at 371–388 kcal of energy. With similar protein contents, our results suggested that *A. viridiflora* provided less fat with higher carbohydrate and fiber contents (Table 2, Table 3, Table 4 and Table 5). Our results also suggested that young shoots of *A. viridiflora* provided higher contents of most nutrients such as protein, fat, and dietary fiber than its old leaves. These results corresponded to a previous report on moringas, detailing the fact that young leaves exhibited higher protein contents than old leaves [14]. Opposite results were observed for mineral contents. In most plants, including *A. viridiflora*, mineral contents increased as the leaves matured [14,15]. Interestingly, zinc contents in young leaves of many plants were found to be higher than in old leaves [14,16], with similar results recorded in *A. viridiflora*. Old leaves of *A. viridiflora* gave higher vitamin C contents than young shoots, with similar results reported in mature and immature leaves of the spider plant (*Cleome gynandra*) and black nightshade (*Solanum* spp.) [17]. However, vitamin C contents of mature and immature leaves depended on the particular plant. A previous study found that vitamin C contents decreased in *Moringa oleifera* leaves as they aged [18], while sugar (glucose and fructose) contents in young leaves of *Arabidopsis thaliana* were higher than in older leaves under warm conditions [19], with opposite results revealed under cold temperature [19].

Old leaves of *A. viridiflora* also exhibited higher total phenolic contents (TPCs) than young shoots. Similar results for TPCs were observed in leaves of *Moringa oleifera*, *Clausena lansium* and *Carthamus tinctorius* [18,20,21]. However, TPCs were also shown to be dependent on particular plants. Young leaves of *Camilla sinensis*, *Schima superba*, *Cryptocarya concinna*, and *Ilex paraguariensis* exhibited higher TPCs than their mature leaves [22,23,24]. Interestingly, total flavonoid contents (TFCs) in some plants produced the opposite result to TPCs. For example, old leaves of *Clausena lansium* exhibited higher TFCs than young leaves, even though the latter provided higher TFCs than the former [20]. Flavonoids are a sub-class of phenolics and a major biological function of phenolics is as antioxidants. An unclear trend on TPCs and TFCs led to ambiguous antioxidant activities. Our results suggested higher antioxidant activities in old leaves than in young shoots of *A. viridiflora*. Similar results were found in *Moringa oleifera*, where DPPH radical scavenging activities increased as the leaves aged [18]. However, higher total antioxidant capacity (TAC) was observed in immature leaves of *Schima superba* and *Cryptocarya concinna* than their mature leaves [23].

The effect of seasonal variation suggested that *A. viridiflora* harvested in July–August (end of harvesting period, high rainfall) had high nutritional composition, while March–April (beginning of harvesting period, low rainfall with monsoon season in late April) was suitable for harvesting the plant with high phenolics. Our results concurred with previous reports on other plants. The autumn (high total rainfall) harvest of *Medicago sativa* L. from Saudi Arabia provided high human nutrition (high crude protein and fat but low crude fiber) [25], while protein contents in *Acacia brevispica*, *Acacia nilotica*, *Acacia seyal*, *Acacia tortilis*, *Balanites aegyptiaca*, *Grewia bicolor*, *Grewia tembensis*, and *Rhus natalensis* from Southern Ethiopia harvested during the rainy season were higher than recorded in the hot dry season [26]. Similar results were observed in *Cladium mariscus* L. Pohl (sawgrass) from Southern Portugal, where crude protein and fat contents were higher in spring than during other seasons, while fiber was lowest [27]. Phenolics in *Secondatia floribunda* A. DC. were higher in the dry season than during the rainy season [28]. Phenolic contents in sawgrass were reported to be high in summer, while the lowest value was found in spring [27]. However, no significant seasonal differences in antioxidant activities determined by DPPH and FRAP assays were detected [27]. Similar results were observed in *Secondatia floribunda* [28]. After a long resting period (September–February), *A. viridiflora* produced high secondary metabolites (i.e., phenolics) to deter herbivores and microbes that might otherwise interfere with its growth and survival, as well as serve as growth factors [29,30]. 

Lastly, all plant origins exhibited varied nutritional compositions, phenolic contents, and antioxidant activities. The impact of geographical origin on these variables remains unclear even though principal component analysis and agglomerative hierarchical clustering analysis suggested that seasonal variation had less impact on MN. Thus, the MN cultivar was determined as the most suitable for future sample quality control. Further plant genomic studies should be conducted to confirm these findings.

To be concluded, nutrients including energy, protein, fat, dietary fiber, phosphorus, sodium, and zinc were high in young shoots, while old leaves favored in total sugar, vitamin C, carotenoids, and some minerals including potassium, calcium, magnesium, and iron. Old leaves also exhibited high phenolic contents and most antioxidant activities. Interestingly, the early harvesting period (March–April) of *A. viridiflora* yielded high phenolics, while the late harvesting period (July–August) was appropriate for high nutrients. However, the effect of plant origins on most nutrients, phenolic contents, and antioxidant activities was unclear. This research demonstrated useful information regarding how nutritive values, phenolics, and antioxidant activities of *A. viridiflora* are affected by plant part, plant origin, and seasonal climate variations. Knowledge gained from this research provides useful agricultural information to promote the production and consumption of *A. viridiflora* as a potentially healthy plant food.

## Figures and Tables

**Table 1:** Statistics of rainfall at meteorology station (M.38C) in Sikhio district, Nakhon Ratchasima province, Thailand.

Harvesting Times (Month/2018)	Rainfall (mm)
March	7.8
April	289.0
May	214.8
June	41.3
July	67.7
August	94.9

Statistics of rainfall was received from Lower Northeastern Region Hydrological lrrigation Center, Bureau of Water Management and Hydrology, Royal Irrigation Department, Thailand (http://hydro-4.rid.go.th (accessed on 16 March 2021)).

**Table 2:** Nutritional compositions including energy, protein, fat, carbohydrate and ash (per 100 g dry weight) in young shoots and old leaves of *Adenia viridiflora* Craib. from different origins including Kamphaeng Phet (KP), Muang Nakhon Ratchasima (MN), Pakchong Nakhon Ratchasima (PN), and Uthai Thani (UT) collected in different harvesting periods.

Nutrients	Young Shoots			Old Leaves		
	March–April	May–June	July–August	March–April	May–June	July–August
**Energy (kcal)**						
KP	381.52 ± 4.36 ^aB^	387.69 ± 0.08 ^aA^ *	381.06 ± 0.28 ^cB^ *	380.80 ± 2.27 ^bA^	379.72 ± 0.40 ^aA^	380.17 ± 4.06 ^abA^
MN	382.50 ± 1.47 ^aA^ *	376.99 ± 0.93 ^cB^	381.92 ± 1.41 ^bcA^	373.22 ± 0.68 ^cB^	376.08 ± 1.67 ^aAB^	376.79 ± 1.90 ^bA^
PN	376.42 ± 0.24 ^bC^ *	381.52 ± 0.79 ^bB^ *	386.55 ± 0.51 ^aA^	386.97 ± 0.73 ^aA^	376.31 ± 1.16 ^aC^	382.95 ± 2.80 ^aB^
UT	371.45 ± 0.11 ^cC^ *	373.95 ± 0.97 ^dB^	382.76 ± 0.56 ^bA^ *	382.44 ± 1.24 ^bA^	376.66 ± 3.32 ^aB^	379.72 ± 0.04 ^abAB^
**Protein (g)**						
KP	20.82 ± 0.36 ^aA^	19.33 ± 0.06 ^bB^ *	18.74 ± 0.48 ^bB^	21.22 ± 0.13 ^bA^	17.19 ± 0.06 ^cC^	19.48 ± 0.29 ^aB^
MN	20.73 ± 0.72 ^aA^	20.04 ± 0.29 ^bA^ *	18.43 ± 0.21 ^bB^	19.85 ± 0.28 ^cA^	18.19 ± 0.19 ^bB^	18.02 ± 0.63 ^bB^
PN	18.15 ± 0.18 ^cC^ *	21.94 ± 0.22 ^aA^ *	20.87 ± 0.24 ^aB^ *	22.68 ± 0.29 ^aA^	19.67 ± 0.26 ^aB^	17.80 ± 0.25 ^bC^
UT	19.66 ± 0.00 ^bAB^ *	19.15 ± 1.20 ^bC^ *	20.76 ± 0.48 ^aA^ *	18.98 ± 0.28 ^dA^	16.53 ± 0.45 ^dB^	19.21 ± 0.40 ^aA^
**Fat (g)**						
KP	2.43 ± 0.71 ^aB^	4.10 ± 0.14 ^aA^ *	3.14 ± 0.14 ^aB^	2.29 ± 0.48 ^bA^	3.28 ± 0.19 ^aA^	2.91 ± 0.73 ^aA^
MN	2.35 ± 0.15 ^aA^ *	2.01 ± 0.08 ^cB^	2.61 ± 0.24 ^bA^	1.66 ± 0.19 ^cA^	2.31 ± 0.29 ^bA^	2.24 ± 0.64 ^aA^
PN	0.62 ± 0.07 ^bC^ *	2.77 ± 0.05 ^bB^ *	3.27 ± 0.00 ^aA^	3.09 ± 0.11 ^aA^	2.13 ± 0.20 ^bB^	2.66 ± 0.43 ^aAB^
UT	0.64 ± 0.04 ^bC^ *	2.08 ± 0.28 ^cB^	3.14 ± 0.23 ^aA^ *	2.69 ± 0.18 ^abA^	1.82 ± 0.72 ^bA^	2.23 ± 0.06 ^aA^
**Carbohydrate (g)**						
KP	69.09 ± 0.13 ^cA^	68.36 ± 0.36 ^abA^ *	69.47 ± 0.85 ^bA^	68.82 ± 0.64 ^bB^	70.35 ± 0.28 ^bA^	69.02 ± 0.35 ^bB^
MN	69.60 ± 0.68 ^cB^	69.68 ± 0.34 ^aB^ *	71.18 ± 0.02 ^aA^	69.72 ± 0.54 ^aA^	70.62 ± 0.43 ^bA^	71.13 ± 1.60 ^aA^
PN	74.56 ± 0.28 ^aA^ *	67.21 ± 0.54 ^cC^ *	68.42 ± 0.12 ^bcB^ *	67.12 ± 0.35 ^cC^	69.63 ± 0.10 ^bB^	71.94 ± 0.03 ^aA^
UT	71.76 ± 0.06 ^bA^ *	69.65 ± 1.59 ^aB^ *	67.87 ± 0.85 ^cB^ *	70.57 ± 0.18 ^aB^	73.53 ± 1.24 ^aA^	70.69 ± 0.55 ^aB^
**Ash (g)**						
KP	7.66 ± 0.21 ^bC^	8.21 ± 0.16 ^bB^ *	8.66 ± 0.24 ^aA^	7.67 ± 0.03 ^bC^	9.17 ± 0.14 ^aA^	8.59 ± 0.10 ^aB^
MN	7.31 ± 0.18 ^cC^ *	8.27 ± 0.13 ^bA^ *	7.79 ± 0.05 ^cB^ *	8.77 ± 0.06 ^aA^	8.87 ± 0.05 ^bA^	8.61 ± 0.33 ^aA^
PN	6.67 ± 0.03 ^dC^ *	8.08 ± 0.27 ^bA^ *	7.44 ± 0.12 ^dB^	7.11 ± 0.05 ^cC^	8.58 ± 0.04 ^cA^	7.59 ± 0.16 ^bB^
UT	7.94 ± 0.02 ^aC^ *	9.12 ± 0.11 ^aA^ *	8.23 ± 0.15 ^bB^ *	7.75 ± 0.08 ^bB^	8.12 ± 0.07 ^dA^	7.86 ± 0.09 ^bB^

All data were expressed as mean ± standard deviation (SD) of triplicate experiments (*n* = 3). Small letters indicate significant different values (*p* < 0.05) in the same plant parts of different originated *A. viridiflora* Craib. collected from the same harvesting periods, while capital letters indicate significant different values (*p* < 0.05) in the same plant parts of the same originated *A. viridiflora* Craib. collected from different harvesting periods using one-way analysis of variance (ANOVA) and Duncan’s multiple comparison test; * indicates significant different values (*p* < 0.05) between young shoot and old leaves of the same originated *A. viridiflora* Craib. collected from the same harvesting period using unpaired *t*-test.

**Table 3:** Dietary fiber and sugar contents (per 100 g dry weight) in young shoots and old leaves of *Adenia viridiflora* Craib. from different origins including Kamphaeng Phet (KP), Muang Nakhon Ratchasima (MN), Pakchong Nakhon Ratchasima (PN), and Uthai Thani (UT) collected in different harvesting periods.

Nutrients	Young Shoots			Old Leaves		
	March–April	May–June	July–August	March–April	May–June	July–August
**Total dietary fiber (g)**						
KP	36.16 ± 0.90 ^cB^ *	36.54 ± 0.97 ^dB^ *	58.16 ± 2.06 ^aA^ *	29.15 ± 0.38 ^cC^	42.89 ± 0.40 ^bB^	51.46 ± 0.99 ^aA^
MN	32.89 ± 0.41 ^dB^ *	49.97 ± 0.59 ^bA^ *	48.57 ± 2.50 ^bA^	34.39 ± 0.09 ^bB^	44.60 ± 0.47 ^abA^	48.10 ± 3.85 ^abA^
PN	43.98 ± 1.39 ^bB^ *	40.98 ± 0.68 ^cB^ *	50.75 ± 2.63 ^bA^	39.32 ± 1.29 ^aC^	42.93 ± 0.48 ^bB^	46.42 ± 1.19 ^bA^
UT	60.46 ± 1.28 ^aA^ *	65.26 ± 4.06 ^aA^ *	52.11 ± 0.55 ^bB^ *	28.37 ± 0.12 ^cB^	46.43 ± 1.80 ^aA^	46.49 ± 0.98 ^bA^
**Soluble dietary fiber (g)**						
KP	5.91 ± 1.22 ^cB^	11.84 ± 0.16 ^bA^ *	10.39 ± 0.86 ^abA^	4.32 ± 0.13 ^dC^	10.64 ± 0.66 ^cB^	12.86 ± 1.45 ^aA^
MN	3.43 ± 0.01 ^dB^ *	9.52 ± 0.12 ^cA^ *	8.85 ± 1.53 ^bA^ *	6.25 ± 0.09 ^bC^	12.50 ± 0.52 ^bB^	14.58 ± 1.70 ^aA^
PN	12.44 ± 0.32 ^bA^ *	10.83 ± 1.42 ^bcA^ *	12.10 ± 1.19 ^aA^	11.20 ± 0.16 ^aB^	18.96 ± 0.67 ^aA^	12.58 ± 1.38 ^aB^
UT	15.19 ± 1.45 ^aB^ *	20.47 ± 1.69 ^aA^	11.88 ± 1.74 ^aC^	4.91 ± 0.09 ^cC^	17.93 ± 1.26 ^aA^	12.27 ± 0.31 ^aB^
**Insoluble dietary fiber (g)**						
KP	30.25 ± 0.32 ^cB^ *	24.70 ± 1.13 ^dC^ *	47.77 ± 1.20 ^aA^ *	24.82 ± 0.25 ^bC^	32.25 ± 0.26 ^aB^	38.60 ± 0.46 ^aA^
MN	29.46 ± 0.42 ^cB^ *	40.45 ± 0.47 ^bA^ *	39.72 ± 0.97 ^bA^ *	28.14 ± 0.00 ^aB^	32.11 ± 0.06 ^aA^	33.52 ± 2.15 ^bA^
PN	31.53 ± 1.07 ^bB^ *	30.14 ± 0.74 ^cB^ *	38.65 ± 1.43 ^bA^ *	28.12 ± 1.13 ^aB^	23.97 ± 0.19 ^cC^	33.84 ± 0.18 ^bA^
UT	45.27 ± 0.17 ^aA^ *	44.79 ± 2.37 ^aA^ *	40.23 ± 1.19 ^bB^ *	23.46 ± 0.21 ^cC^	28.50 ± 0.53 ^bB^	34.22 ± 1.29 ^bA^
**Total sugar (g)**						
KP	11.49 ± 0.25 ^aB^ *	12.11 ± 0.35 ^bB^ *	15.00 ± 0.45 ^bA^ *	15.15 ± 0.07 ^aB^	13.44 ± 0.70 ^bC^	18.04 ± 0.06 ^aA^
MN	8.98 ± 0.23 ^cB^ *	8.45 ± 0.05 ^dC^ *	16.02 ± 0.31 ^aA^ *	13.96 ± 0.20 ^bB^	14.66 ± 0.27 ^aB^	18.19 ± 0.69 ^aA^
PN	8.51 ± 0.19 ^dC^ *	10.04 ± 0.49 ^cB^ *	14.89 ± 0.03 ^bA^ *	11.83 ± 0.11 ^dC^	13.64 ± 0.05 ^abB^	18.22 ± 0.57 ^aA^
UT	10.81 ± 0.14 ^bC^ *	14.60 ± 0.16 ^aB^	16.16 ± 0.08 ^aA^	13.57 ± 0.16 ^cB^	13.53 ± 0.85 ^bB^	16.60 ± 0.54 ^bA^
**Fructose (g)**						
KP	2.02 ± 0.12 ^aC^ *	4.04 ± 0.04 ^bA^ *	3.37 ± 0.11 ^bB^ *	5.16 ± 0.28 ^aB^	4.89 ± 0.09 ^cB^	6.21 ± 0.08 ^aA^
MN	2.09 ± 0.17 ^aC^ *	4.03 ± 0.14 ^bB^ *	4.48 ± 0.13 ^aA^ *	4.32 ± 0.01 ^bC^	5.49 ± 0.12 ^aB^	6.44 ± 0.23 ^aA^
PN	1.50 ± 0.00 ^bC^ *	3.83 ± 0.19 ^bA^ *	3.06 ± 0.00 ^cB^ *	4.58 ± 0.18 ^bB^	4.81 ± 0.01 ^cAB^	5.03 ± 0.12 ^bA^
UT	0.88 ± 0.07 ^cC^ *	4.62 ± 0.11 ^aA^ *	3.24 ± 0.05 ^bB^ *	4.50 ± 0.10 ^bB^	5.15 ± 0.00 ^bA^	5.34 ± 0.20 ^bA^
**Glucose (g)**						
KP	9.47 ± 0.12 ^bA^	8.06 ± 0.31 ^bB^	9.71 ± 0.25 ^bA^	9.44 ± 0.18 ^aA^	7.48 ± 0.49 ^aB^	9.89 ± 0.13 ^aA^
MN	6.88 ± 0.06 ^cB^ *	4.42 ± 0.19 ^dC^ *	9.92 ± 0.16 ^bA^	7.66 ± 0.20 ^cC^	8.30 ± 0.12 ^aB^	10.15 ± 0.42 ^aA^
PN	7.01 ± 0.19 ^cB^	6.21 ± 0.68 ^cB^ *	9.98 ± 0.12 ^bA^	7.25 ± 0.08 ^dC^	8.05 ± 0.09 ^aB^	10.11 ± 0.45 ^aA^
UT	9.93 ± 0.07 ^aB^ *	9.98 ± 0.28 ^aB^ *	10.88 ± 0.11 ^aA^ *	9.07 ± 0.26 ^bAB^	8.38 ± 0.86 ^aB^	9.52 ± 0.26 ^aA^
**Sucrose (g)**						
KP	ND	ND	1.91 ± 0.08 ^b^	0.55 ± 0.02 ^bC^	1.08 ± 0.13 ^aB^	1.94 ± 0.01 ^bA^
MN	<LOD	ND	1.62 ± 0.02 ^c^	1.98 ± 0.01 ^aA^	0.87 ± 0.03 ^bC^	1.60 ± 0.04 ^dB^
PN	<LOD	ND	1.86 ± 0.08 ^b^ *	ND	0.78 ± 0.03 ^bB^	3.09 ± 0.01 ^aA^
UT	<LOD	ND	2.04 ± 0.02 ^a^ *	<LOD	ND	1.74 ± 0.08 ^cA^

All data were expressed as mean ± standard deviation (SD) of triplicate experiments (*n* = 3). Small letters indicate significant different values (*p* < 0.05) in the same plant parts of different originated *A. viridiflora* Craib. collected from the same harvesting periods, while capital letters indicate significant different values (*p* < 0.05) in the same plant parts of the same originated *A. viridiflora* Craib. collected from different harvesting periods using one-way analysis of variance (ANOVA) and Duncan’s multiple comparison test; * indicates significant different values (*p* < 0.05) between young shoot and old leaves of the same originated *A. viridiflora* Craib. collected from the same harvesting period using an unpaired *t*-test. ND: not detected. ND: not detected; LOD: limit of detection (0.02 g/100 g).

**Table 4:** Vitamin C and carotenoid (lutein and β-carotene) contents (per 100 g dry weight) in young shoots and old leaves of *Adenia viridiflora* Craib. from different origins including Kamphaeng Phet (KP), Muang Nakhon Ratchasima (MN), Pakchong Nakhon Ratchasima (PN), and Uthai Thani (UT) collected in different harvesting periods.

Nutrients	Young Shoots			Old Leaves		
	March–April	May–June	July–August	March–April	May–June	July–August
**Vitamin C (mg)**						
KP	754.27 ± 28.67 ^bC^ *	1034.83 ± 26.26 ^bB^ *	1292.44 ± 99.87 ^bA^ *	1058.37 ± 37.71 ^bC^	1349.45 ± 0.35 ^bB^	1841.90 ± 28.65 ^bA^
MN	776.90 ± 51.55 ^bC^ *	1019.73 ± 41.34 ^bB^ *	1311.54 ± 2.98 ^bA^ *	862.64 ± 1.27 ^dC^	1105.31 ± 12.07 ^dB^	1598.29 ± 53.21 ^cA^
PN	573.93 ± 23.69 ^cC^ *	1231.19 ± 79.40 ^aA^	800.81 ± 72.52 ^cB^ *	928.17 ± 8.76 ^cC^	1200.02 ± 32.83 ^cB^	1501.99 ± 20.89 ^dA^
UT	879.40 ± 10.81 ^aC^ *	1007.39 ± 56.25 ^bB^ *	1696.46 ± 18.22 ^aA^ *	1181.70 ± 6.03 ^aC^	1439.03 ± 42.55 ^aB^	2240.65 ± 39.79 ^aA^
**Lutein (mg)**						
KP	65.20 ± 0.21 ^aA^ *	55.29 ± 0.39 ^aB^ *	48.74 ± 3.24 ^aC^ *	93.49 ± 3.22 ^aA^	80.00 ± 3.22 ^aB^	79.74 ± 3.89 ^aB^
MN	49.75 ± 0.07 ^bA^ *	47.94 ± 3.57 ^bA^ *	31.00 ± 3.12 ^bB^ *	84.61 ± 2.94 ^bA^	82.82 ± 3.13 ^aA^	64.10 ± 4.42 ^bB^
PN	33.25 ± 1.55 ^dA^ *	26.47 ± 1.67 ^dC^ *	30.44 ± 0.73 ^bB^ *	62.43 ± 4.25 ^dB^	70.12 ± 0.17 ^bA^	50.26 ± 2.62 ^cC^
UT	38.10 ± 0.47 ^cB^ *	41.32 ± 1.00 ^cA^ *	33.92 ± 2.50 ^bC^ *	69.30 ± 3.83 ^cAB^	66.04 ± 1.28 ^bB^	75.09 ± 3.91 ^aA^
**β-carotene** **(mg)**						
KP	27.24 ± 0.91 ^aA^ *	20.14 ± 1.32 ^aB^ *	18.65 ± 1.19 ^aB^ *	47.04 ± 2.67 ^aA^	41.71 ± 1.57 ^bAB^	37.19 ± 3.47 ^aB^
MN	20.78 ± 0.09 ^bA^ *	18.50 ± 1.22 ^aA^ *	12.89 ± 0.84 ^bC^ *	45.77 ± 1.28 ^aA^	44.46 ± 0.91 ^bA^	33.70 ± 2.52 ^aB^
PN	19.36 ± 1.58 ^bA^ *	13.94 ± 0.90 ^bB^ *	18.08 ± 0.86 ^aA^ *	44.74 ± 3.07 ^aB^	49.84 ± 0.53 ^aA^	35.28 ± 1.55 ^aC^
UT	14.51 ± 0.01 ^cA^ *	14.49 ± 0.25 ^bA^ *	12.08 ± 1.00 ^bB^ *	34.39 ± 2.18 ^bA^	31.52 ± 2.59 ^cA^	34.99 ± 0.91 ^aA^

All data were expressed as mean ± standard deviation (SD) of triplicate experiments (*n* = 3). Small letters indicate significant different values (*p* < 0.05) in the same plant parts of different originated *A. viridiflora* Craib. collected from the same harvesting periods, while capital letters indicate significant different values (*p* < 0.05) in the same plant parts of the same originated *A. viridiflora* Craib. collected from different harvesting periods using one-way analysis of variance (ANOVA) and Duncan’s multiple comparison test; * indicates significant different values (*p* < 0.05) between young shoot and old leaves of the same originated *A. viridiflora* Craib. collected from the same harvesting period using an unpaired *t*-test.

**Table 5:** Minerals including calcium, phosphorus, sodium, potassium, magnesium, iron, and zinc (per 100 g dry weight) in young shoots and old leaves of *Adenia viridiflora* Craib. from different origins including Kamphaeng Phet (KP), Muang Nakhon Ratchasima (MN), Pakchong Nakhon Ratchasima (PN), and Uthai Thani (UT) collected in different harvesting periods.

Nutrients	Young Shoots			Old Leaves		
	March–April	May–June	July–August	March–April	May–June	July–August
**Calcium (mg)**						
KP	653.93 ± 29.03 ^aC^ *	920.61 ± 4.37 ^aA^ *	854.27 ± 29.36 ^aB^ *	1137.28 ± 4.22 ^bC^	1645.10 ± 63.78 ^aA^	1277.89 ± 17.38 ^bB^
MN	669.84 ± 1.44 ^aB^ *	895.96 ± 40.42 ^aA^ *	907.11 ± 19.55 ^aA^ *	1610.80 ± 23.08 ^aA^	1642.38 ± 105.65 ^aA^	1438.75 ± 83.01 ^aB^
PN	397.97 ± 0.22 ^bC^ *	631.55 ± 37.25 ^cA^ *	529.35 ± 25.74 ^cB^ *	968.40 ± 11.87 ^cC^	1554.59 ± 14.57 ^aA^	1165.90 ± 19.29 ^cB^
UT	418.83 ± 7.81 ^bC^ *	786.85 ± 70.01 ^bA^ *	621.94 ± 36.62 ^bB^ *	977.39 ± 15.34 ^cB^	1371.91 ± 26.54 ^bA^	1000.70 ± 33.63 ^dB^
**Phosphorus (mg)**						
KP	534.08 ± 71.72 ^aA^	601.59 ± 82.19 ^aA^	561.18 ± 36.40 ^aA^ *	450.88 ± 10.80 ^bB^	477.53 ± 6.97 ^aA^	433.28 ± 1.29 ^bC^
MN	477.13 ± 19.34 ^aA^ *	512.81 ± 6.65 ^aA^ *	467.56 ± 34.41 ^bA^	418.16 ± 11.73 ^cA^	401.93 ± 11.60 ^bA^	428.15 ± 19.11 ^bA^
PN	531.45 ± 4.85 ^aA^ *	535.66 ± 59.77 ^aA^ *	579.87 ± 0.55 ^aA^ *	472.77 ± 3.33 ^aA^	418.90 ± 18.29 ^bB^	421.54 ± 17.10 ^bB^
UT	537.39 ± 57.99 ^aA^	555.34 ± 48.21 ^aA^ *	557.51 ± 9.87 ^aA^ *	477.52 ± 11.11 ^aA^	432.89 ± 28.95 ^bB^	459.07 ± 3.83 ^aAB^
**Sodium (mg)**						
KP	135.42 ± 50.46 ^aA^	132.56 ± 26.68 ^aA^ *	102.97 ± 55.61 ^aA^	62.39 ± 12.25 ^abB^	86.01 ± 1.16 ^bA^	59.71 ± 0.20 ^bB^
MN	55.11 ± 3.16 ^bA^	86.23 ± 45.18 ^aA^	51.74 ± 7.64 ^aA^	74.86 ± 37.24 ^aA^	61.59 ± 16.87 ^bA^	48.68 ± 5.15 ^cA^
PN	120.22 ± 9.21 ^aAB^ *	152.02 ± 43.54 ^aA^	87.49 ± 11.24 ^aA^	52.32 ± 12.15 ^abC^	131.97 ± 2.97 ^aA^	103.75 ± 0.60 ^aB^
UT	95.99 ± 38.10 ^abA^ *	93.04 ± 33.50 ^aA^	69.43 ± 1.85 ^aA^ *	30.65 ± 4.48 ^bA^	66.94 ± 32.50 ^bA^	44.30 ± 7.59 ^cA^
**Potassium (mg)**						
KP	2077.47 ± 93.61 ^aB^ *	1949.93 ± 26.25 ^bB^ *	2356.72 ± 79.16 ^bA^ *	1543.54 ± 7.56 ^bB^	1543.54 ± 8.89 ^cB^	2001.22 ± 71.09 ^bA^
MN	1810.88 ± 73.40 ^bB^ *	2186.31 ± 71.34 ^bA^ *	2177.13 ± 39.18 ^cA^ *	1357.16 ± 81.06 ^cB^	1877.61 ± 162.93 ^aA^	1983.55 ± 51.67 ^bA^
PN	1740.09 ± 66.25 ^bC^ *	2197.43 ± 8.66 ^bB^ *	2443.19 ± 28.34 ^bA^ *	1497.68 ± 33.61 ^bcB^	1709.12 ± 34.03 ^bA^	1587.42 ± 107.86 ^cAB^
UT	2177.00 ± 79.72 ^aB^ *	2475.16 ± 267.19 ^aAB^ *	2549.33 ± 50.89 ^aA^ *	1852.70 ± 133.06 ^aB^	1741.04 ± 23.68 ^abB^	2144.11 ± 8.72 ^aA^
**Magnesium (mg)**						
KP	307.49 ± 21.15 ^bB^ *	217.54 ± 1.97 ^bC^ *	473.54 ± 31.55 ^aA^ *	463.15 ± 6.33 ^bB^	341.69 ± 9.35 ^aC^	762.89 ± 15.67 ^bA^
MN	357.48 ± 16.83 ^aB^ *	298.00 ± 22.65 ^aC^ *	450.54 ± 10.53 ^abA^ *	539.60 ± 6.68 ^aB^	344.00 ± 17.27 ^aC^	951.84 ± 31.55 ^aA^
PN	242.92 ± 4.00 ^cB^ *	247.40 ± 2.70 ^bB^ *	378.99 ± 17.32 ^cA^ *	427.70 ± 3.43 ^cB^	355.33 ± 1.72 ^aC^	601.00 ± 7.97 ^cA^
UT	245.26 ± 11.72 ^cB^ *	236.51 ± 21.54 ^bB^ *	428.42 ± 7.40 ^bA^ *	394.97 ± 4.37 ^dB^	305.46 ± 2.69 ^bC^	798.77 ± 20.61 ^bA^
**Iron (mg)**						
KP	4.84 ± 0.27 ^aB^ *	4.28 ± 0.32 ^cC^ *	6.06 ± 0.06 ^aA^ *	6.24 ± 0.01 ^bB^	6.49 ± 0.12 ^bAB^	7.51 ± 0.89 ^aA^
MN	4.66 ± 0.03 ^abB^ *	5.19 ± 0.04 ^bA^ *	4.93 ± 0.29 ^cAB^ *	8.57 ± 1.24 ^aA^	7.08 ± 0.12 ^aAB^	6.43 ± 0.63 ^abB^
PN	4.27 ± 0.11 ^bC^ *	7.08 ± 0.39 ^aA^	5.63 ± 0.14 ^bB^	6.83 ± 0.08 ^bA^	7.29 ± 0.44 ^aA^	5.71 ± 0.18 ^bB^
UT	4.39 ± 0.31 ^bB^ *	5.08 ± 0.36 ^bA^	5.49 ± 0.25 ^bA^	6.35 ± 0.26 ^bA^	4.90 ± 0.20 ^cB^	5.84 ± 0.46 ^bA^
**Zinc (mg)**						
KP	3.80 ± 0.27 ^aA^ *	3.76 ± 0.06 ^aA^	3.89 ± 0.17 ^aA^ *	2.99 ± 0.12 ^aC^	3.90 ± 0.15 ^aA^	3.46 ± 0.01 ^aB^
MN	2.25 ± 0.01 ^cC^	2.63 ± 0.17 ^bB^	3.19 ± 0.03 ^cA^ *	2.60 ± 0.27 ^bB^	2.59 ± 0.17 ^dB^	3.54 ± 0.07 ^aA^
PN	3.13 ± 0.12 ^bB^	3.68 ± 0.10 ^aA^	3.79 ± 0.04 ^aA^ *	2.93 ± 0.11 ^aB^	3.44 ± 0.25 ^bA^	3.14 ± 0.20 ^bAB^
UT	3.17 ± 0.45 ^bB^	3.85 ± 0.17 ^aA^ *	3.50 ± 0.09 ^bAB^ *	2.59 ± 0.07 ^bB^	3.07 ± 0.02 ^cA^	3.00 ± 0.11 ^bA^

All data were expressed as mean ± standard deviation (SD) of triplicate experiments (*n* = 3). Small letters indicate significant different values (*p* < 0.05) in the same plant parts of different originated *A. viridiflora* Craib. collected from the same harvesting periods, while capital letters indicate significant different values (*p* < 0.05) in the same plant parts of the same originated *A. viridiflora* Craib. collected from different harvesting periods using one-way analysis of variance (ANOVA) and Duncan’s multiple comparison test; * indicates significant different values (*p* < 0.05) between young shoot and old leaves of the same originated *A. viridiflora* Craib. collected from the same harvesting period using an unpaired *t*-test.

**Table 6:** Total phenolic contents (TPCs), total flavonoid contents (TFCs), and antioxidant activities in young shoots and old leaves of *Adenia viridiflora* Craib. from different origins including Kamphaeng Phet (KP), Muang Nakhon Ratchasima (MN), Pakchong Nakhon Ratchasima (PN), and Uthai Thani (UT) collected in different harvesting periods.

Activities	Young Shoots			Old Leaves		
	March–April ^#^	May–June	July–August	March–April ^#^	May–June	July–August
**Total phenolic contents (mg GAE/g DW)**						
KP	20.57 ± 0.72 ^cB^ *	18.94 ± 1.20 ^cC^ *	26.95 ± 1.81 ^aA^	28.70 ± 1.79 ^aA^	23.71 ± 1.05 ^cB^	23.84 ± 1.99 ^bB^
MN	26.35 ± 1.84 ^aA^	22.47 ± 1.25 ^aB^ *	20.79 ± 1.41 ^bcC^ *	25.37 ± 1.16 ^bA^	24.86 ± 0.93 ^bA^	24.38 ± 1.43 ^bA^
PN	23.57 ± 0.92 ^bA^ *	20.59 ± 1.92 ^bB^ *	21.39 ± 0.97 ^bB^ *	27.89 ± 1.61 ^aA^	24.80 ± 0.64 ^bB^	25.70 ± 2.02 ^bB^
UT	20.38 ± 0.79 ^cB^ *	21.50 ± 0.48 ^abA^ *	19.69 ± 1.21 ^cB^ *	23.56 ± 1.34 ^cB^	28.05 ± 1.44 ^aA^	27.97 ± 2.44 ^aA^
**Total flavonoid contents (mg QE/g DW)**						
KP	7.35 ± 0.42 ^bB^ *	5.42 ± 0.54 ^bC^ *	10.91 ± 0.92 ^aA^	5.96 ± 0.35 ^cC^	8.42 ± 0.72 ^bB^	10.84 ± 0.88 ^bA^
MN	7.15 ± 0.50 ^bB^ *	5.37 ± 0.52 ^bC^ *	8.78 ± 0.83 ^cA^ *	8.35 ± 0.61 ^bC^	9.32 ± 0.46 ^aB^	11.92 ± 0.64 ^aA^
PN	9.65 ± 0.72 ^aA^ *	6.24 ± 0.37 ^aB^ *	9.98 ± 0.87 ^bA^ *	14.08 ± 1.41 ^aA^	8.44 ± 0.31 ^bC^	12.61 ± 0.52 ^aB^
UT	7.50 ± 0.69 ^bB^ *	6.24 ± 0.59 ^aC^ *	8.53 ± 0.64 ^cA^ *	8.38 ± 0.33 ^bB^	7.60 ± 0.63 ^cC^	9.38 ± 0.77 ^cA^
**2,2-Diphenyl-1-picrylhydrazyl (DPPH)** **radical scavenging activities (µmol TE/100 g DW)**						
KP	1.25 ± 0.06 ^bA^ *	0.98 ± 0.08 ^aC^	1.15 ± 0.02 ^aB^ *	0.96 ± 0.07 ^bA^	1.02 ± 0.09 ^aA^	1.03 ± 0.05 ^bA^
MN	1.37 ± 0.04 ^aA^ *	0.86 ± 0.07 ^bC^ *	1.02 ± 0.07 ^bB^ *	0.99 ± 0.06 ^bB^	1.02 ± 0.10 ^aB^	1.16 ± 0.02 ^aA^
PN	1.28 ± 0.02 ^bA^ *	0.95 ± 0.03 ^aC^ *	1.02 ± 0.04 ^bB^ *	1.44 ± 0.10 ^aA^	1.04 ± 0.08 ^aC^	1.12 ± 0.06 ^aB^
UT	1.23 ± 0.10 ^bA^ *	0.78 ± 0.06 ^cC^ *	1.03 ± 0.08 ^bB^ *	0.97 ± 0.05 ^bC^	1.05 ± 0.09 ^aB^	1.14 ± 0.09 ^aA^
**Ferric reducing antioxidant power (FRAP)** **activities (µmol TE/g DW)**						
KP	16.52 ± 0.97 ^bC^ *	20.20 ± 1.73 ^bB^ *	25.63 ± 1.64 ^aA^ *	34.08 ± 3.12 ^bB^	39.83 ± 3.99 ^aA^	30.72 ± 0.88 ^cC^
MN	29.24 ± 2.88 ^aA^ *	22.76 ± 2.22 ^aB^ *	19.47 ± 1.68 ^cC^ *	35.74 ± 3.27 ^abA^	34.91 ± 3.19 ^bA^	34.20 ± 2.10 ^bA^
PN	28.03 ± 0.98 ^aA^ *	22.73 ± 1.43 ^aB^ *	22.42 ± 1.59 ^bB^ *	37.00 ± 1.60 ^aB^	40.31 ± 3.41 ^aA^	37.71 ± 3.33 ^aAB^
UT	14.36 ± 0.85 ^cC^ *	19.82 ± 1.87 ^bA^ *	17.06 ± 1.50 ^dB^ *	35.17 ± 2.36 ^abB^	42.90 ± 1.68 ^aA^	27.08 ± 2.18 ^dC^
**Oxygen radical absorbance capacity (ORAC)** **activities (µmol TE/g DW)**						
KP	1148.47 ± 74.70 ^bA^ *	945.45 ± 80.39 ^bB^	843.16 ± 26.88 ^bC^ *	753.77 ± 74.81 ^cB^	878.30 ± 71.10 ^bA^	557.60 ± 12.22 ^bC^
MN	842.36 ± 69.59 ^cA^ *	819.31 ± 74.57 ^cA^ *	879.40 ± 73.51 ^abA^	1094.66 ± 119.33 ^bA^	979.12 ± 88.65 ^aB^	817.34 ± 70.76 ^bC^
PN	1438.85 ± 145.27 ^aA^	1264.75 ± 83.41 ^aB^ *	913.14 ± 58.62 ^aC^ *	1403.53 ± 122.44 ^aA^	933.96 ± 66.54 ^abB^	592.08 ± 20.22 ^cC^
UT	1066.29 ± 100.98 ^bB^	1338.09 ± 81.11 ^aA^ *	649.06 ± 58.15 ^cC^ *	1106.77 ± 102.36 ^bB^	916.29 ± 73.94 ^abC^	1209.10 ± 75.07 ^aA^

All data were expressed as mean ± standard deviation (SD) of triplicate experiments (*n* = 3). GAE: gallic acid equivalent; QE: quercetin equivalent; TE: trolox equivalent; DW: dry weight; small letters indicate significant different values (*p* < 0.05) in the same plant parts of different originated *A. viridiflora* Craib. collected from the same harvesting periods, while capital letters indicate significant different values (*p* < 0.05) in the same plant parts of the same originated *A. viridiflora* Craib. collected from different harvesting periods using one-way analysis of variance (ANOVA) and Duncan’s multiple comparison test; * indicates significant different values (*p* < 0.05) between young shoot and old leaves of the same originated *A. viridiflora* Craib. collected from the same harvesting period using an unpaired *t*-test. ^#^ Information from the previous report [2].

## Data Availability

Data are contained within this article and Supplementary Material.

## Supplementary Materials

The following are available online at https://www.mdpi.com/article/10.3390/foods10112799/s1: Supplementary Table S1. Images of young shoots of Kamphaeng Phet (KP), Muang Nakhon Ratchasima (MN), Pakchong Nakhon Ratchasima (PN), and Uthai Thani (UT) originated *Adenia viridiflora* Craib. collected from different harvesting periods; Supplementary Table S2. Images of old leaves of Kamphaeng Phet (KP), Muang, Nakhon Ratchasima (MN), Pakchong, Nakhon Ratchasima (PN), and Uthai Thani (UT) originated *Adenia viridiflora* Craib. collected from different harvesting periods; Supplementary Table S3. Color analysis of fresh and dried young shoots and old leaves of Kamphaeng Phet (KP), Muang, Nakhon Ratchasima (MN), Pakchong, Nakhon Ratchasima (PN), and Uthai Thani (UT) originated *Adenia viridiflora* Craib. collected from different harvesting periods; Supplementary Table S4. The moisture contents of fresh and dried young shoots and old leaves of Kamphaeng Phet (KP), Muang, Nakhon Ratchasima (MN), Pakchong, Nakhon Ratchasima (PN), and Uthai Thani (UT) originated *Adenia viridiflora* Craib from different harvesting periods; Supplementary Table S5. Nutritional compositions (per 100 g fresh weight) in young shoots and old leaves of *Adenia viridiflora* Craib. collected from Kamphaeng Phet (KP) origin in different harvesting periods; Supplementary Table S6. Nutritional compositions (per 100 g fresh weight) in young shoots and old leaves of *Adenia viridiflora* Craib. collected from Muang Nakhon Ratchasima (MN) origin in different harvesting periods; Supplementary Table S7. Nutritional compositions (per 100 g fresh weight) in young shoots and old leaves of *Adenia viridiflora* Craib. collected from Pakchong Nakhon Ratchasima (PN) origin in different harvesting periods; Supplementary Table S8. Nutritional compositions (per 100 g fresh weight) in young shoots and old leaves of *Adenia viridiflora* Craib. collected from Uthai Thani (UT) origin in different harvesting periods.

## References

[1] Polito L., Bortolotti M., Maiello S., Battelli M.G., Bolognesi A. (2016). Plants producing robosome-inactivateing proteins in traditional medicine. Molecules.

[2] Wannasaksri W., On-Nom N., Chupeerach C., Temviriyanukul P., Charoenkiatkul S., Suttisansanee U. (2021). In Vitro Phytotherapeutic Properties of Aqueous Extracted *Adenia viridiflora* Craib. towards Civilization Diseases. Molecules.

[3] Latimer G.W. (2019). Official Method of Analysis of AOAC International.

[4] Chupeerach C., Aursalung A., Watcharachaisoponsiri T., Whanmek K., Thiyajai P., Yosphan K., Sritalahareuthai V., Sahasakul Y., Santivarangkna C., Suttisansanee U. (2021). The Effect of Steaming and Fermentation on Nutritive Values, Antioxidant Activities, and Inhibitory Properties of Tea Leaves. Foods.

[5] Hinkaew J., Aursalung A., Sahasakul Y., Tangsuphoom N., Suttisansanee U. (2021). A Comparison of Nutritional and Biochemical Quality of Date Palm Fruits Obtained from Different Planting Techniques. Molecules.

[6] Sritalahareuthai V., Aursalung A., On-Nom N., Temviriyanukul P., Charoenkiatkul S., Suttisansanee U. (2020). Nutritional composition of conserved Kadsura spp. plants in Northern Thailand. Heliyon.

[7] Thuphairo K., Sornchan P., Suttisansanee U. (2019). Bioactive Compounds, Antioxidant Activity and Inhibition of Key Enzymes Relevant to Alzheimer’s Disease from Sweet Pepper (*Capsicum annuum*) Extracts. Prev. Nutr. Food Sci..

[8] Suttisansanee U., Thiyajai P., Chalermchaiwat P., Wongwathanarat K., Pruesapan K., Charoenkiatkul S., Temviriyanukul P. (2021). Phytochemicals and In Vitro Bioactivities of Aqueous Ethanolic Extracts from Common Vegetables in Thai Food. Plants.

[9] Zhishen J., Mengcheng T., Jianming W. (1999). The determination of flavonoid contents in mulberry and their scavenging effects on superoxide radicals. Food Chem..

[10] Sripum C., Kukreja R.K., Charoenkiatkul S., Kriengsinyos W., Suttisansanee U. (2017). The effect of extraction conditions on antioxidant activities and total phenolic contents of different processed Thai Jasmine rice. Int. Food Res. J..

[11] Magdalene O.A., Okpashi V.E., Bayim B.P. (2019). Comparative Evaluation of Proximate Composition of Selected Wild-edible Plants in Central Cross River State. J. Sci. Eng. Technol..

[12] Nnamani C.V., Oselebe H.O., Agbatutu A. (2009). Assessment of nutritional values of three underutilized indigenous leafy vegetables of Ebonyi State, Nigeria. Afr. J. Biotechnol..

[13] Maroyi A. (2020). Evaluation of Medicinal uses, Phytochemistry and Biological Activities of *Adenia gummifera* (Harv.) Harms. J. Pharm. Nutr. Sci..

[14] Salim A., Hasyim M., Adam A. (2018). Nutrient Contents of Moringa Leaves based on Leaf Age. Indian J. Public Health Res. Dev..

[15] Maillard A., Diquélou S., Billard V., Laîné P., Garnica M., Prudent M., Garcia-Mina J.-M., Yvin J.-C., Ourry A. (2015). Leaf mineral nutrient remobilization during leaf senescence and modulation by nutrient deficiency. Front. Plant Sci..

[16] Flyman M.V., Afolayan A.J. (2008). Effect of plant maturity on the mineral content of the leaves of *Monordica balsamina* L. and *Vigna unguiculata* subsp. *sesquipedalis* (L.) Verdc. J. Food Qual..

[17] Ayua E., Mugalavai V., Simon J., Weller S., Obura P., Nyabinda N. (2016). Ascorbic acid content in leaves of Nightshade (*Solanum* spp.) and spider plant (*Cleome gynandra*) varieties grown under different fertilizer regimes in Western Kenya. Afr. J. Biotechnol..

[18] Nobossé P., Fombang E.N., Mbofung C.M.F. (2018). Effects of age and extraction solvent on phytochemical content and antioxidant activity of fresh *Moringa oleifera* L. leaves. Food Sci. Nutr..

[19] Wingler A., Stangberg E.J., Saxena T., Mistry R. (2012). Interactions Between Temperature and Sugars in the Regulation of Leaf Senescence in the Perennial Herb *Arabis alpina* L.. J. Integr. Plant Biol..

[20] Chang X., Lu Y., Lin Z., Qiu I., Guo X., Pan J., Abbasi A.M. (2018). Impact of Leaf Development Stages on Polyphenolics Profile and Antioxidant Activity in *Clausena lansium* (Lour.) Skeels. BioMed Res. Int..

[21] Abdallah S.B., Rabhi M., Harbaoui F., Zar-kalai F., Lachâal M., Karray-Bouraoui N. (2013). Distribution of phenolic compounds and antioxidant activity between young and old leaves of *Carthamus tinctorius* L. and their induction by salt stress. Acta Physiol. Plant.

[22] Liu Z., Bruins M.E., de Bruijn W.J.C., Vincken J. (2020). A comparison of the phenolic composition of old and young tea leaves reveals a decrease in flavanols and phenolic acids and an increase in flavonols upon tea leaf maturation. J. Food Compost. Anal..

[23] Zhang T., Zheng J., Yu Z., Huang X., Zhang Q., Tian X., Peng C. (2018). Functional characteristics of phenolic compounds accumulated in young leaves of two subtropical forest tree species of different successional stages. Tree Physiol..

[24] Blum-Silva C.H., Chaves V.C., Schenkel E.P., Coelho G.C., Reginatto F.H. (2015). The influence of leaf age on methylxanthines, total phenolic content, and free radical scavenging capacity of *Ilex paraguariensis* aqueous extracts. Rev. Bras Farmacogn..

[25] Soufan W., Okla M.K., Salamatullah A., Hayat K., Abdel-Maksoud M.A., Al-Amri S.S. (2021). Seasonal variation in yield, nutritive value, and antioxidant capacity of leaves of alfalfa plants grown in arid climate of Saudi Arabia. Chil. J. Agric. Res..

[26] Abebe A., Tolera A., Holand Ø., Ådnøy T., Eik L.O. (2012). Seasonal variation in nutritive value of some browse and grass species in Borane rangeland, southern Ethiopia. Trop. Subtrop. Agroecosyst..

[27] Oliveira M., João Rodrigues M., Neng N.R., Nogueira J.M.F., Bessa R.J.B., Custódio L. (2021). Seasonal Variations of the Nutritive Value and Phytotherapeutic Potential of *Cladium mariscus* L. (Pohl.) Targeting Ruminant’s Production. Plants.

[28] Ribeiro D.A., Camilo C.J., de Fátima Alves Nonato C., Rodrigues F.F.G., Menezes I.R.A., Ribeiro-Filho J., Xiao J., de Almeida Souza M.M., da Costa J.G.M. (2020). Influence of seasonal variation on phenolic content and in vitro antioxidant activity of *Secondatia floribunda* A. DC. (Apocynaceae). Food Chem..

[29] Wink M. (2008). Plant Secondary Metabolism: Diversity, Function and its Evolution. Nat. Prod. Commun..

[30] Demain A.L., Fang A. (2000). The natural functions of secondary metabolites. Adv. Biochem. Eng. Biotechnol..

