# Supplementary material for: Influence of Plant Origins and Seasonal Variations on Nutritive Values, Phenolics and Antioxidant Activities of Adenia viridiflora Craib., an Endangered Species from Thailand
Source: Foods. 2021 Nov 14;10(11):2799. doi: 10.3390/foods10112799 (PMC8623237; doi:10.3390/foods10112799)
Supplement: Supplementary file 1 [file foods-10-02799-s001.zip › foods-1452523-supplementary.pdf]

Supplementary materials

Influence of Plant Origins and Seasonal Variations on Nutritive Values, Phenolics and Antioxidant Activities of *Adenia viridiflora* Craib., an Endangered Species from Thailand

Werawat Wannasaksri [1], Piya Temviriyanukul [1], Amornrat Aursalung [1], Yuraporn Sahasakul [1], Sirinapa Thangsiri [1], Woorawee Inthachat [1], Nattira On-Nom [1], Chaowanee Chupeerach [1], Kanchana Pruesapan [2], Somsri Charoenkiatkul [1] and Uthaiwan Suttisansanee [1,*]

[1] Institute of Nutrition, Mahidol University, Salaya, Phuttamonthon, Nakhon Pathom 73170, Thailand; nit.frank@gmail.com (W.W.); piya.tem@mahidol.ac.th (P.T.); amornrat.aur@mahidol.ac.th (A.A.); yuraporn.sah@mahidol.ac.th (Y.S.); sirinapa.thang@outlook.com (S.T.); woorawee.int@mahidol.ac.th (W.I.); nattira.onn@mahidol.ac.th (N.O.); chaowanee.chu@mahidol.ac.th (C.C.); somsri.chr@mahidol.ac.th (S.C.); uthaiwan.sut@mahidol.ac.th (U.S.)

[2] Plant Varieties Protection Division, Department of Agriculture, Ministry of Agriculture and Cooperatives, Bangkok 10900, Thailand; kpruesapan@gmail.com (K.P.)

* Correspondence: uthaiwan.sut@mahidol.ac.th; Tel.: +66-(0)-2800-2380 (ext. 422)

Supplementary Table S1:

Images of young shoots of Kamphaeng Phet (KP), Muang Nakhon Ratchasima (MN), Pakchong Nakhon Ratchasima (PN), and Uthai Thani (UT) originated *Adenia viridiflora* Craib. collected from different harvesting periods.

**Origins**	**Harvesting periods**		
	**March-April**	**May-June**	**July-August**
KP	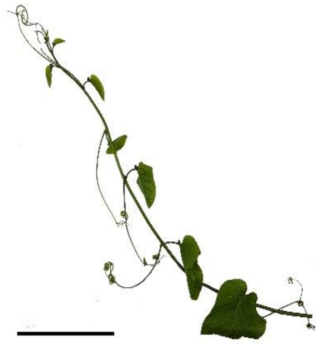	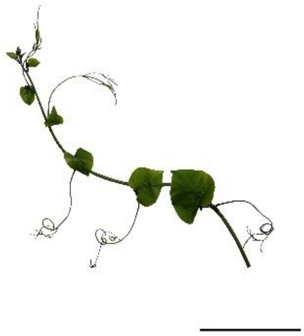	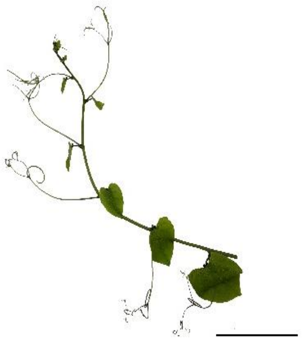
MN	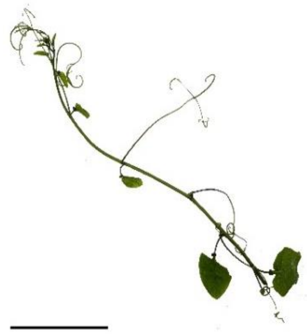	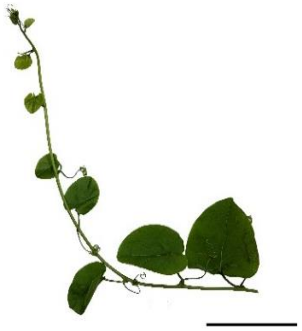	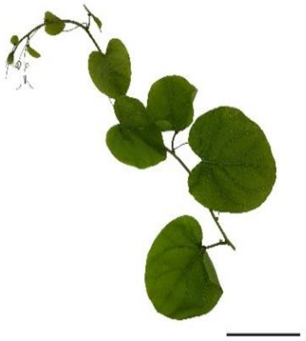
PN	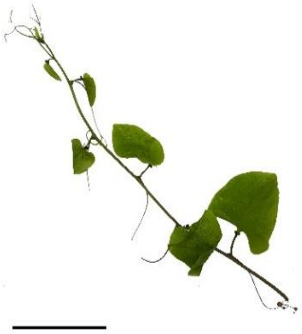	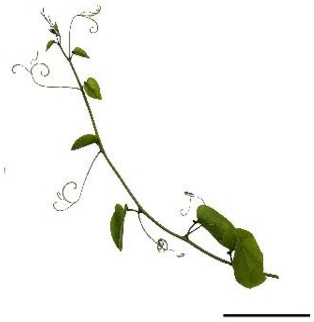	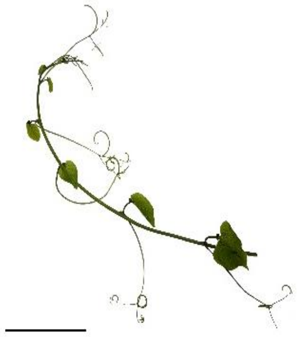
UT	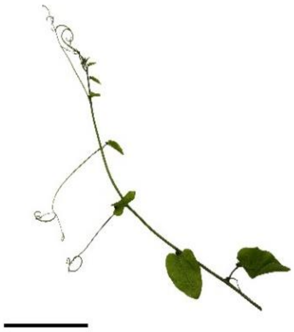	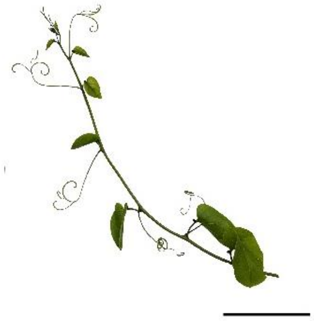	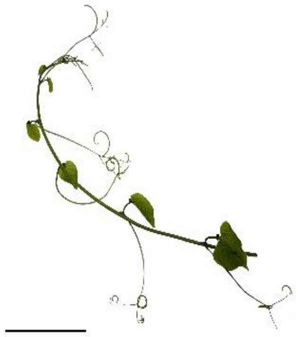

Note: – scale of 10 cm.

Supplementary Table S2:

Images of old leaves of Kamphaeng Phet (KP), Muang, Nakhon Ratchasima (MN), Pakchong, Nakhon Ratchasima (PN), and Uthai Thani (UT) originated *Adenia viridiflora* Craib. collected from different harvesting periods.

**Origins**	**Harvesting periods**		
	**March-April**	**May-June**	**July-August**
KP	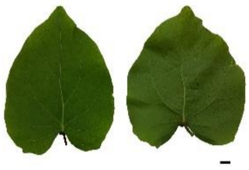	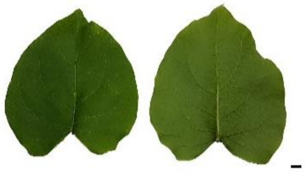	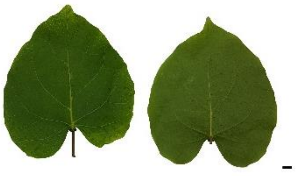
MN	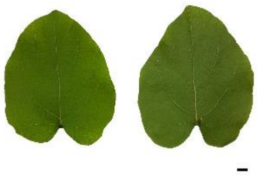	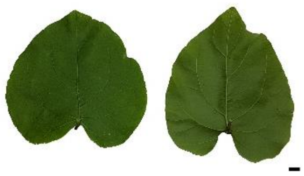	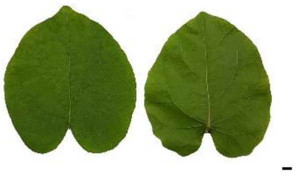
PN	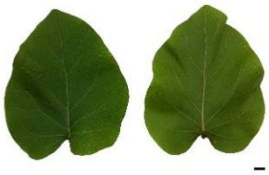	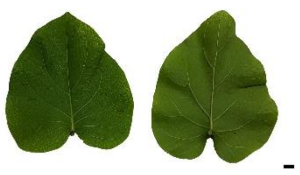	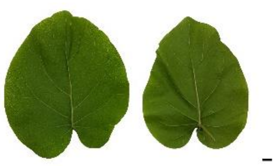
UT	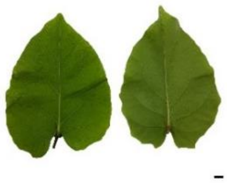	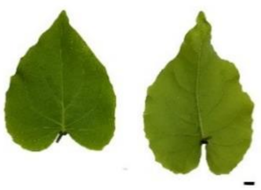	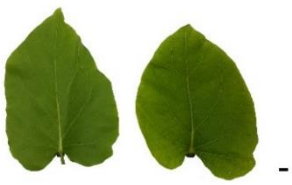

Note: – scale of 1 cm.

Supplementary Table S3:

Color analysis of fresh and dried young shoots and old leaves of Kamphaeng Phet (KP), Muang, Nakhon Ratchasima (MN), Pakchong, Nakhon Ratchasima (PN), and Uthai Thani (UT) originated *Adenia viridiflora* Craib. collected from different harvesting periods.

Samples	Color values of fresh samples			Color values of dried samples		
	**L***	**a***	**b***	**L***	**a***	**b***
**Young shoots from March-April harvesting periods**						
KP	34.50 ± 1.70	-6.49 ± 0.89	22.52 ± 1.07	36.86 ± 0.63	-4.97 ± 0.27	14.07 ± 0.84
MN	32.27 ± 4.61	-5.92 ± 0.89	21.51 ± 1.50	37.48 ± 0.26	-5.02 ± 0.02	14.79 ± 0.35
PN	41.89 ± 1.43	-8.87 ± 0.57	26.71 ± 1.91	39.05 ± 0.43	-5.52 ± 0.10	16.82 ± 0.23
UT	35.96 ± 4.69	-7.19 ± 2.95	24.60 ± 4.40	38.90 ± 0.17	-4.90 ± 0.13	15.07 ± 0.36
**Young shoots from May-June harvesting periods**						
KP	34.61 ± 5.08	-7.17 ± 1.55	21.64 ± 2.01	37.18 ± 1.46	-4.50 ± 0.43	13.79 ± 1.33
MN	40.57 ± 3.68	-8.84 ± 091	24.84 ± 1.34	37.18 ± 0.23	-3.55 ± 2.53	13.52 ± 019
PN	36.49 ± 2.02	-8.56 ± 0.36	25.50 ± 1.27	38.51 ± 1.80	-4.12 ± 0.55	13.47 ± 1.25
UT	36.67 ± 5.73	-8.27 ± 0.98	26.96 ± 3.04	39.27 ± 0.17	-4.62 ± 0.50	15.04 ± 1.47
**Young shoots from July-August harvesting periods**						
KP	38.80 ± 6.44	-8.02 ± 2.52	25.87 ± 4.17	38.54 ± 2.93	-5.06 ± 0.54	15.69 ± 2.05
MN	37.67 ± 4.02	-7.57 ± 1.11	23.43 ± 2.68	38.90 ± 1.23	-5.08 ± 0.25	15.72 ± 0.57
PN	41.49 ± 3.11	-9.06 ± 0.20	28.51 ± 3.84	39.24 ± 1.07	-4.49 ± 0.34	14.43 ± 0.53
UT	33.19 ± 2.43	-7.11 ± 1.21	24.62 ± 2.49	42.39 ± 0.50	-5.23 ± 0.40	17.24 ± 1.10
**Old leaves from March-April harvesting periods**						
KP	42.92 ± 1.53	-9.66 ± 0.73	25.38 ± 1.11	35.37 ± 0.52	-5.14 ± 0.08	14.61 ± 0.41
MN	47.00 ± 2.39	-9.47 ± 0.02	26.94 ± 2.05	35.17 ± 0.98	-5.19 ± 0.21	14.85 ± 0.80
PN	46.12 ± 0.55	-10.02 ± 0.20	28.12 ± 1.47	35.48 ± 1.19	-4.81 ± 1.07	14.36 ± 1.75
UT	46.80 ± 3.80	-9.29 ± 0.51	29.65 ± 1.42	32.34 ± 0.11	-3.87 ± 0.06	11.95 ± 0.04
**Old leaves from May-June harvesting periods**						
KP	42.83 ± 4.14	-9.03 ± 0.12	24.96 ± 1.63	34.30 ± 1.91	-4.88 ± 0.66	13.80 ± 1.96
MN	39.95 ± 1.81	-8.49 ± 0.69	23.67 ± 0.23	43.59 ± 6.80	-5.60± 0.06	16.07 ± 0.95
PN	41.87 ± 2.99	-9.28 ± 0.40	25.43 ± 3.80	34.36 ± 0.97	-5.12 ± 0.38	13.69 ± 0.93
UT	40.63 ± 0.86	-10.13 ± 0.24	24.97 ± 1.93	35.83 ± 0.11	-5.28 ± 0.15	15.10 ± 0.27
**Old leaves from July-August harvesting periods**						
KP	44.47 ± 1.76	-9.83 ± 0.13	27.91 ± 0.63	33.89 ± 0.36	-3.76 ± 0.53	12.28 ± 0.87
MN	45.62 ± 2.38	-9.44 ± 0.30	28.25 ± 2.76	37.45 ± 0.38	-5.39 ± 0.26	15.99 ± 0.36
PN	44.56 ± 2.18	-9.42 ± 0.37	28.58 ± 2.21	35.92 ± 1.53	-4.69 ± 1.26	14.35 ± 2.11
UT	44.47 ± 6.98	-9.75 ± 1.06	31.94 ± 5.90	37.29 ± 0.84	-5.11 ± 0.25	15.69 ± 0.19

All data were expressed as mean ± standard deviation (SD) of triplicate experiments ($n = 3$). L* describes darkness (−) to lightness (+), a* describes green (−) to red (+) colors, and b* describes indigo (−) to yellow (+).

Supplementary Table S4:

The moisture contents of fresh and dried young shoots and old leaves of Kamphaeng Phet (KP), Muang, Nakhon Ratchasima (MN), Pakchong, Nakhon Ratchasima (PN), and Uthai Thani (UT) originated *Adenia viridiflora* Craib from different harvesting periods.

Varieties	Moisture content (%)		
	**March-April**	**May-June**	**July-August**
**Fresh young shoots**			
KP	85.78 ± 0.40	83.95 ± 0.51	84.65 ± 0.52
MN	83.99 ± 0.37	83.69 ± 0.20	84.26 ± 0.46
PN	85.47 ± 0.30	84.37 ± 0.32	85.48 ± 0.32
UT	86.87 ± 0.77	84.69 ± 0.25	85.61 ± 0.41
**Fresh old leaves**			
KP	80.17 ± 0.17	78.00 ± 0.67	81.07 ± 0.23
MN	80.43 ± 0.67	78.01 ± 0.26	80.04 ± 0.67
PN	82.11 ± 0.51	79.60 ± 0.08	81.22 + 0.06
UT	80.91 ± 0.57	81.62 ± 0.53	83.08 ± 0.17
**Dried young shoots**			
KP	6.79 ± 0.83	6.03 ± 1.20	6.15 ± 0.79
MN	5.39 ± 0.95	5.18 ± 0.72	6.61 ± 1.05
PN	7.79 ± 0.31	6.29 ± 1.02	5.32 ± 0.63
UT	6.73 ± 2.30	6.41 ± 0.98	7.21 ± 0.67
**Dried old leaves**			
KP	6.19 ± 0.54	5.54 ± 0.39	5.52 ± 0.46
MN	6.77 ± 0.23	6.92 ± 0.27	6.26 ± 0.19
PN	6.71 ± 0.26	5.64 ± 0.30	6.09 ± 1.19
UT	7.81 ± 1.40	6.71 ± 1.29	7.38 ± 0.38

All data were expressed as mean ± standard deviation (SD) of triplicate experiments (n = 3).

Supplementary Table S5:

Nutritional compositions (per 100 g fresh weight) in young shoots and old leaves of *Adenia viridiflora* Craib. collected from Kamphaeng Phet (KP) origin in different harvesting periods.

Nutrients	Young shoots			Old leaves		
	March-April	May-June	July-August	March-April	May-June	July-August
**Energy (kcal)**	56.85 ± 3.10 ^{A*}	61.86 ± 3.01 ^{A*}	55.98 ± 3.51 ^{A*}	79.70 ± 0.51 ^{a}	79.29 ± 0.74 ^{a}	68.61 ± 1.49 ^{b}
**Moisture (g)**	85.11 ± 0.57 ^{A*}	84.05 ± 0.77 ^{A*}	85.31 ± 0.91 ^{A*}	79.07 ± 0.04 ^{b}	79.12 ± 0.23 ^{b}	81.96 ± 0.12 ^{a}
**Protein (g)**	3.10 ± 0.04 ^{A*}	3.09 ± 0.16 ^{AB*}	2.75 ± 0.07 ^{B*}	4.44 ± 0.03 ^{a}	3.59 ± 0.06 ^{b}	3.52 ± 0.05 ^{b}
**Fat (g)**	0.37 ± 0.16 ^{A}	0.66 ± 0.06 ^{A}	0.46 ± 0.00 ^{A}	0.48 ± 0.14 ^{a}	0.69 ± 0.05 ^{a}	0.53 ± 0.19 ^{a}
**Carbohydrate (g)**	10.29 ± 0.37 ^{A*}	10.91 ± 0.45 ^{A*}	10.21 ± 0.81 ^{A*}	14.41 ± 0.22 ^{a}	14.69 ± 0.24 ^{a}	12.46 ± 0.01 ^{b}
**TDF (g)**	5.39 ± 0.40 ^{B}	5.83 ± 0.06 ^{B*}	8.53 ± 0.10 ^{A*}	6.10 ± 0.10 ^{b}	8.96 ± 0.02 ^{a}	9.29 ± 0.19 ^{a}
- SDF (g)	0.89 ± 0.29 ^{B}	1.89 ± 0.13 ^{A}	1.52 ± 0.08 ^{A*}	0.91 ± 0.04 ^{b}	2.22 ± 0.17 ^{a}	2.32 ± 0.35 ^{a}
- IDF (g)	4.51 ± 0.11 ^{B*}	3.94 ± 0.06 ^{C*}	7.01 ± 0.18 ^{A}	5.20 ± 0.06 ^{b}	6.74 ± 0.15 ^{a}	6.97 ± 0.16 ^{a}
**Total sugar (g)**	1.71 ± 0.01 ^{C*}	1.93 ± 0.01 ^{B*}	2.20 ± 0.04 ^{A*}	3.17 ± 0.03 ^{a}	2.81 ± 0.18 ^{b}	3.26 ± 0.01 ^{a}
- Fructose(g)	0.30 ± 0.01 ^{C*}	0.65 ± 0.02 ^{A*}	0.50 ± 0.01 ^{B*}	1.08 ± 0.08 ^{a}	1.02 ± 0.01 ^{a}	1.12 ± 0.03 ^{a}
- Glucose(g)	1.41 ± 0.03 ^{A*}	1.29 ± 0.01 ^{B*}	1.43 ± 0.04 ^{A*}	1.98 ± 0.05 ^{a}	1.56 ± 0.13 ^{b}	1.79 ± 0.02 ^{ab}
- Sucrose(g)	ND	ND	0.28 ± 0.00 *	0.12 ± 0.01 ^{c}	0.23 ± 0.04 ^{b}	0.35 ± 0.00 ^{a}
**Ash (g)**	1.14 ± 0.00 ^{A*}	1.31 ± 0.10 ^{A*}	1.27 ± 0.03 ^{A*}	1.61 ± 0.01 ^{b}	1.92 ± 0.02 ^{a}	1.55 ± 0.01 ^{c}
**Vitamin C (mg)**	112.47 ± 10.36 ^{B*}	165.25 ± 13.90 ^{A*}	189.22 ± 9.05 ^{A*}	221.51 ± 10.71 ^{c}	281.77 ± 2.95 ^{b}	332.40 ± 9.52 ^{a}
**Minerals (mg)**						
- Calcium	97.29 ± 2.37 ^{C*}	146.86 ± 6.11 ^{A*}	125.31 ± 1.63 ^{B*}	238.04 ± 1.73 ^{b}	343.40 ± 15.11 ^{a}	230.58 ± 2.90 ^{b}
- Phosphorus	79.26 ± 12.05 ^{A}	95.54 ± 13.91 ^{A}	82.21 ± 2.48 ^{A}	94.37 ± 3.01 ^{a}	99.72 ± 3.14 ^{a}	78.19 ± 0.19 ^{b}
- Sodium	20.38 ± 11.41 ^{A}	21.01 ± 5.00 ^{A}	14.77 ± 10.62 ^{A}	13.06 ± 3.60 ^{ab}	17.96 ± 0.54 ^{a}	10.78 ± 0.02 ^{b}
- Potassium	309.06 ± 7.82 ^{A}	311.26 ± 20.95 ^{A}	345.70 ± 4.89 ^{A}	323.07 ± 2.89 ^{a}	322.31 ± 6.12 ^{a}	361.18 ± 20.55 ^{a}
- Magnesium	45.72 ± 2.69 ^{B*}	34.72 ± 2.12 ^{B*}	69.77 ± 10.84 ^{A*}	96.94 ± 1.68 ^{b}	71.36 ± 3.54 ^{c}	137.65 ± 3.08 ^{a}
- Iron	0.72 ± 0.03 ^{A*}	0.69 ± 0.11 ^{A*}	0.89 ± 0.04 ^{A*}	1.31 ± 0.01 ^{a}	1.36 ± 0.05 ^{a}	1.36 ± 0.22 ^{a}
- Zinc	0.57 ± 0.04 ^{A}	0.60 ± 0.04 ^{A*}	0.57 ± 0.00 ^{A*}	0.63 ± 0.04 ^{b}	0.82 ± 0.04 ^{a}	0.63 ± 0.01 ^{b}

All data were expressed as mean ± standard deviation (SD) of triplicate experiments (n = 3). ND: not detected; TDF: total dietary fiber; SDF: soluble dietary fiber; IDF: insoluble dietary fiber; capital and small letters indicate significant differences ($p < 0.05$) of the same nutrients in young shoots and old leaves, respectively, from different harvesting periods using one-way analysis of variance (ANOVA) and Duncan's multiple comparison test; * indicates significant differences ($p < 0.05$) of the same nutrient between young shoot and old leaves from the same harvesting period using unpaired t-test.

Supplementary Table S6:

Nutritional compositions (per 100 g fresh weight) in young shoots and old leaves of *Adenia viridiflora* Craib. collected from Muang Nakhon Ratchasima (MN) origin in different harvesting periods.

Nutrients	Young shoots			Old leaves		
	March-April	May-June	July-August	March-April	May-June	July-August
**Energy (kcal)**	71.44 ± 3.28 ^{A*}	61.81 ± 1.90 ^{B*}	63.54 ± 1.22 ^{B}	82.11 ± 1.85 ^{a}	75.67 ± 0.37 ^{ab}	69.71 ± 3.85 ^{b}
**Moisture (g)**	81.33 ± 0.76 ^{B*}	83.61 ± 0.45 ^{A*}	83.37 ± 0.23 ^{A}	78.00 ± 0.55 ^{b}	79.88 ± 0.23 ^{ab}	81.50 ± 1.15 ^{a}
**Protein (g)**	3.88 ± 0.35 ^{A}	3.29 ± 0.02 ^{AB*}	3.07 ± 0.01 ^{B*}	4.37 ± 0.02 ^{a}	3.66 ± 0.01 ^{b}	3.33 ± 0.04 ^{c}
**Fat (g)**	0.44 ± 0.06 ^{A}	0.33 ± 0.03 ^{A}	0.44 ± 0.06 ^{A}	0.37 ± 0.05 ^{a}	0.47 ± 0.08 ^{a}	0.41 ± 0.14 ^{a}
**Carbohydrate (g)**	13.00 ± 0.35 ^{A*}	11.43 ± 0.39 ^{B*}	11.84 ± 0.17 ^{B}	15.34 ± 0.55 ^{a}	14.21 ± 0.28 ^{a}	13.18 ± 1.24 ^{a}
**TDF (g)**	6.14 ± 0.14 ^{B*}	8.19 ± 0.08 ^{A*}	8.08 ± 0.47 ^{A}	7.57 ± 0.16 ^{b}	8.98 ± 0.23 ^{a}	8.87 ± 0.45 ^{a}
- SDF (g)	0.64 ± 0.03 ^{B*}	1.56 ± 0.01 ^{A*}	1.47 ± 0.34 ^{A*}	1.38 ± 0.01 ^{b}	2.52 ± 0.18 ^{a}	2.69 ± 0.28 ^{a}
- IDF (g)	5.50 ± 0.11 ^{B*}	6.63 ± 0.07 ^{A}	6.61 ± 0.13 ^{A*}	6.19 ± 0.16 ^{a}	6.46 ± 0.06 ^{a}	6.19 ± 0.18 ^{a}
**Total sugar (g)**	1.68 ± 0.01 ^{B*}	1.39 ± 0.05 ^{C*}	2.67 ± 0.04 ^{A*}	3.07 ± 0.01 ^{b}	2.95 ± 0.04 ^{c}	3.36 ± 0.03 ^{a}
- Fructose(g)	0.39 ± 0.03 ^{C*}	0.66 ± 0.01 ^{B*}	0.75 ± 0.02 ^{A*}	0.95 ± 0.03 ^{c}	1.11 ± 0.02 ^{b}	1.19 ± 0.01 ^{a}
- Glucose(g)	1.29 ± 0.04 ^{B*}	0.73 ± 0.06 ^{C*}	1.65 ± 0.01 ^{A*}	1.69 ± 0.02 ^{b}	1.67 ± 0.01 ^{b}	1.88 ± 0.01 ^{a}
- Sucrose(g)	<LOD	ND	0.27 ± 0.00 *	0.44 ± 0.01 ^{a}	0.18 ± 0.01 ^{c}	0.30 ± 0.01 ^{b}
**Ash (g)**	1.37 ± 0.01 ^{A*}	1.36 ± 0.01 ^{A*}	1.30 ± 0.01 ^{B*}	1.93 ± 0.03 ^{a}	1.79 ± 0.04 ^{b}	1.59 ± 0.01 ^{c}
**Vitamin C (mg)**	144.81 ± 7.74 ^{C*}	167.06 ± 5.04 ^{B*}	218.18 ± 3.76 ^{A*}	189.78 ± 4.36 ^{c}	222.37 ± 0.93 ^{b}	295.33 ± 4.50 ^{a}
**Minerals (mg)**						
- Calcium	125.09 ± 4.69 ^{B*}	146.77 ± 5.38 ^{A*}	150.87 ± 2.48 ^{A*}	354.29 ± 1.70 ^{a}	330.62 ± 33.78 ^{a}	265.57 ± 5.14 ^{b}
- Phosphorus	89.00 ± 1.50 ^{A}	84.06 ± 0.74 ^{A}	77.84 ± 9.19 ^{A}	91.95 ± 1.34 ^{a}	80.85 ± 2.39 ^{a}	79.39 ± 9.93 ^{a}
- Sodium	10.28 ± 0.42 ^{A}	14.00 ± 10.09 ^{A}	8.60 ± 1.68 ^{A}	16.62 ± 12.00 ^{a}	12.37 ± 4.66 ^{a}	9.05 ± 1.91 ^{a}
- Potassium	337.79 ± 5.69 ^{A}	358.22 ± 6.80 ^{A}	362.23 ± 14.30 ^{A}	298.26 ± 17.73 ^{a}	378.04 ± 50.61 ^{a}	366.64 ± 9.34 ^{a}
- Magnesium	66.67 ± 1.74 ^{A*}	48.79 ± 3.92 ^{B*}	74.97 ± 3.53 ^{A*}	118.69 ± 0.90 ^{b}	69.19 ± 4.14 ^{c}	175.88 ± 2.72 ^{a}
- Iron	0.87 ± 0.03 ^{A*}	0.85 ± 0.01 ^{A*}	0.82 ± 0.06 ^{A*}	1.88 ± 0.34 ^{a}	1.43 ± 0.05 ^{ab}	1.19 ± 0.09 ^{b}
- Zinc	0.42 ± 0.01 ^{B*}	0.43 ± 0.03 ^{B*}	0.53 ± 0.01 ^{A*}	0.57 ± 0.07 ^{a}	0.52 ± 0.04 ^{a}	0.66 ± 0.02 ^{a}

All data were expressed as mean ± standard deviation (SD) of triplicate experiments (n = 3). ND: not detected; TDF: total dietary fiber; SDF: soluble dietary fiber; IDF: insoluble dietary fiber; capital and small letters indicate significant differences ($p < 0.05$) of the same nutrients in young shoots and old leaves, respectively, from different harvesting periods using one-way analysis of variance (ANOVA) and Duncan's multiple comparison test; * indicates significant differences ($p < 0.05$) of the same nutrient between young shoot and old leaves from the same harvesting period using unpaired t-test.

Supplementary Table S7:

Nutritional compositions (per 100 g fresh weight) in young shoots and old leaves of *Adenia viridiflora* Craib. collected from Pakchong Nakhon Ratchasima (PN) origin in different harvesting periods.

Nutrients	Young shoots			Old leaves		
	**March-April**	**May-June**	**July-August**	**March-April**	**May-June**	**July-August**
**Energy (kcal)**	61.28 ± 1.54 ^{A*}	59.19 ± 2.06 ^{AB*}	55.03 ± 0.80 ^{B*}	73.97 ± 0.17 ^{a}	74.34 ± 0.51 ^{a}	73.17 ± 2.19 ^{a}
**Moisture (g)**	83.72 ± 0.42 ^{B*}	84.49 ± 0.59 ^{AB*}	85.77 ± 0.23 ^{A*}	80.89 ± 0.01 ^{a}	80.25 ± 0.05 ^{a}	80.90 ± 0.37 ^{a}
**Protein (g)**	2.96 ± 0.04 ^{B*}	3.41 ± 0.18 ^{A}	2.97 ± 0.00 ^{B*}	4.34 ± 0.08 ^{a}	3.89 ± 0.06 ^{b}	3.40 ± 0.00 ^{c}
**Fat (g)**	0.10 ± 0.01 ^{B*}	0.43 ± 0.03 ^{A}	0.47 ± 0.01 ^{A}	0.59 ± 0.03 ^{a}	0.42 ± 0.06 ^{a}	0.51 ± 0.13 ^{a}
**Carbohydrate (g)**	12.14 ± 0.38 ^{A}	10.43 ± 0.28 ^{B*}	9.74 ± 0.18 ^{B*}	12.83 ± 0.10 ^{b}	13.76 ± 0.06 ^{a}	13.75 ± 0.26 ^{a}
**TDF (g)**	7.16 ± 0.13 ^{B}	6.36 ± 0.09 ^{B*}	7.22 ± 0.41 ^{A*}	7.52 ± 0.35 ^{b}	8.48 ± 0.11 ^{a}	8.87 ± 0.15 ^{a}
- SDF (g)	2.03 ± 0.02 ^{A}	1.68 ± 0.25 ^{A*}	1.72 ± 0.21 ^{A*}	2.14 ± 0.04 ^{b}	3.75 ± 0.18 ^{a}	2.40 ± 0.33 ^{b}
- IDF (g)	5.13 ± 0.11 ^{AB}	4.68 ± 0.34 ^{B}	5.50 ± 0.20 ^{A*}	5.38 ± 0.30 ^{b}	4.74 ± 0.06 ^{b}	6.47 ± 0.18 ^{a}
**Total sugar (g)**	1.39 ± 0.01 ^{C*}	1.56 ± 0.05 ^{B*}	2.12 ± 0.03 ^{A*}	2.26 ± 0.03 ^{c}	2.70 ± 0.02 ^{b}	3.48 ± 0.08 ^{a}
- Fructose(g)	0.25 ± 0.01 ^{C*}	0.60 ± 0.06 ^{A*}	0.44 ± 0.01 ^{B*}	0.88 ± 0.05 ^{a}	0.95 ± 0.00 ^{a}	0.96 ± 0.01 ^{a}
- Glucose(g)	1.14 ± 0.01 ^{B*}	0.96 ± 0.11 ^{B*}	1.42 ± 0.00 ^{A*}	1.39 ± 0.02 ^{c}	1.59 ± 0.03 ^{b}	1.93 ± 0.08 ^{a}
- Sucrose(g)	<LOD	ND	0.27 ± 0.02 *	ND	0.16 ± 0.01 ^{b}	0.59 ± 0.01 ^{a}
**Ash (g)**	1.09 ± 0.02 ^{A*}	1.26 ± 0.11 ^{A*}	1.06 ± 0.04 ^{A*}	1.36 ± 0.01 ^{c}	1.70 ± 0.01 ^{a}	1.45 ± 0.01 ^{b}
**Vitamin C (mg)**	93.37 ± 3.02 ^{B*}	190.69 ± 10.20 ^{A*}	114.12 ± 16.47 ^{B*}	177.42 ± 2.43 ^{c}	237.08 ± 9.77 ^{b}	286.90 ± 0.01 ^{a}
**Minerals (mg)**						
- Calcium	64.79 ± 1.74 ^{B*}	98.14 ± 11.88 ^{A*}	75.31 ± 3.95 ^{AB*}	185.11 ± 3.14 ^{c}	307.11 ± 3.30 ^{a}	222.70 ± 0.84 ^{b}
- Phosphorus	86.51 ± 1.14 ^{A}	82.86 ± 9.97 ^{A}	82.55 ± 1.46 ^{A}	90.37 ± 0.93 ^{a}	82.76 ± 5.32 ^{a}	80.49 ± 3.04 ^{a}
- Sodium	19.60 ± 2.63 ^{A**}	23.41 ± 8.66 ^{A}	12.44 ± 2.06 ^{A*}	10.00 ± 3.28 ^{c}	26.07 ± 0.76 ^{a}	19.82 ± 0.23 ^{b}
- Potassium	283.49 ± 22.63 ^{B}	340.90 ± 11.00 ^{A}	347.84 ± 11.41 ^{A}	286.28 ± 8.98 ^{b}	337.63 ± 8.66 ^{a}	302.99 ± 23.19 ^{ab}
- Magnesium	39.56 ± 1.95 ^{B*}	38.40 ± 2.04 ^{B*}	53.92 ± 2.60 ^{A*}	81.76 ± 0.90 ^{b}	70.20 ± 0.30 ^{c}	114.80 ± 0.10 ^{a}
- Iron	0.70 ± 0.01 ^{B*}	1.10 ± 0.13 ^{A*}	0.80 ± 0.01 ^{B*}	1.31 ± 0.02 ^{ab}	1.44 ± 0.13 ^{a}	1.09 ± 0.03 ^{b}
- Zinc	0.51 ± 0.01 ^{C}	0.57 ± 0.00 ^{A*}	0.54 ± 0.00 ^{B}	0.56 ± 0.03 ^{a}	0.68 ± 0.07 ^{a}	0.60 ± 0.04 ^{a}

All data were expressed as mean ± standard deviation (SD) of triplicate experiments (n = 3). ND: not detected; TDF: total dietary fiber; SDF: soluble dietary fiber; IDF: insoluble dietary fiber; capital and small letters indicate significant differences ($p < 0.05$) of the same nutrients in young shoots and old leaves, respectively, from different harvesting periods using one-way analysis of variance (ANOVA) and Duncan's multiple comparison test; * indicates significant differences ($p < 0.05$) of the same nutrient between young shoot and old leaves from the same harvesting period using unpaired t-test.

Supplementary Table S8:

Nutritional compositions (per 100 g fresh weight) in young shoots and old leaves of *Adenia viridiflora* Craib. collected from Uthai Thani (UT) origin in different harvesting periods.

Nutrients	Young shoots			Old leaves		
	**March-April**	**May-June**	**July-August**	**March-April**	**May-June**	**July-August**
**Energy (kcal)**	54.98 ± 0.50 ^{A*}	50.68 ± 4.44 ^{A*}	52.57 ± 1.87 ^{A*}	75.98 ± 2.21 ^{a}	69.86 ± 1.63 ^{b}	65.45 ± 1.38 ^{b}
**Moisture (g)**	85.20 ± 0.14 ^{A*}	86.45 ± 1.24 ^{A*}	86.27 ± 0.52 ^{A*}	80.14 ± 0.49 ^{b}	81.45 ± 0.66 ^{ab}	82.77 ± 0.36 ^{a}
**Protein (g)**	2.91 ± 0.03 ^{A*}	2.59 ± 0.01 ^{C*}	2.85 ± 0.01 ^{B*}	3.77 ± 0.01 ^{a}	3.07 ± 0.01 ^{c}	3.31 ± 0.03 ^{b}
**Fat (g)**	0.10 ± 0.01 ^{C*}	0.28 ± 0.03 ^{B}	0.43 ± 0.03 ^{A}	0.54 ± 0.06 ^{a}	0.34 ± 0.18 ^{a}	0.39 ± 0.01 ^{a}
**Carbohydrate (g)**	10.62 ± 0.11 ^{A*}	9.46 ± 1.17 ^{A*}	9.33 ± 0.52 ^{A*}	14.02 ± 0.40 ^{a}	13.65 ± 0.81 ^{ab}	12.19 ± 0.39 ^{b}
**TDF (g)**	8.95 ± 0.35 ^{A*}	8.81 ± 0.03 ^{A}	7.16 ± 0.16 ^{B*}	5.64 ± 0.11 ^{c}	8.61 ± 0.16 ^{a}	8.01 ± 0.07 ^{b}
- SDF (g)	2.25 ± 0.33 ^{AB*}	2.76 ± 0.07 ^{A*}	1.63 ± 0.28 ^{B*}	0.98 ± 0.05 ^{c}	3.32 ± 0.21 ^{a}	2.12 ± 0.12 ^{b}
- IDF (g)	6.70 ± 0.03 ^{A*}	6.05 ± 0.10 ^{AB*}	5.53 ± 0.44 ^{B*}	4.66 ± 0.06 ^{c}	5.29 ± 0.05 ^{b}	5.90 ± 0.19 ^{a}
**Total sugar (g)**	1.60 ± 0.01 ^{B*}	1.98 ± 0.21 ^{AB*}	2.22 ± 0.10 ^{A*}	2.70 ± 0.02 ^{ab}	2.51 ± 0.13 ^{a}	2.86 ± 0.07 ^{a}
- Fructose(g)	0.13 ± 0.01 ^{C*}	0.63 ± 0.04 ^{A*}	0.45 ± 0.01 ^{B*}	0.90 ± 0.05 ^{a}	0.96 ± 0.04 ^{a}	0.92 ± 0.03 ^{a}
- Glucose(g)	1.47 ± 0.00 ^{A*}	1.36 ± 0.18 ^{A}	1.50 ± 0.08 ^{A}	1.80 ± 0.03 ^{a}	1.55 ± 0.17 ^{a}	1.64 ± 0.03 ^{a}
- Sucrose(g)	<LOD	ND	0.28 ± 0.01 *	<LOD	ND	0.30 ± 0.01 ^{a}
**Ash (g)**	1.18 ± 0.01 ^{A*}	1.24 ± 0.09 ^{A*}	1.13 ± 0.01 ^{A*}	1.54 ± 0.01 ^{a}	1.51 ± 0.04 ^{a}	1.36 ± 0.01 ^{b}
**Vitamin C (mg)**	130.14 ± 1.02 ^{B*}	136.06 ± 1.68 ^{B*}	233.08 ± 12.30 ^{A*}	234.77 ± 7.46 ^{c}	266.74 ± 1.60 ^{b}	386.08 ± 1.62 ^{a}
**Minerals (mg)**						
- Calcium	62.00 ± 2.23 ^{C*}	106.05 ± 3.68 ^{A*}	85.29 ± 3.90 ^{B*}	194.11 ± 0.46 ^{b}	254.37 ± 2.16 ^{a}	172.39 ± 4.59 ^{c}
- Phosphorus	79.48 ± 11.38 ^{A}	74.86 ± 2.37 ^{A}	76.61 ± 4.79 ^{A}	94.82 ± 0.79 ^{a}	80.17 ± 4.72 ^{b}	79.13 ± 2.59 ^{b}
- Sodium	14.25 ± 8.11 ^{A}	12.91 ± 7.57 ^{A}	9.53 ± 0.00 ^{A}	6.11 ± 1.41 ^{a}	12.27 ± 8.08 ^{a}	7.62 ± 1.69 ^{a}
- Potassium	322.28 ± 19.76 ^{A}	333.17 ± 20.59 ^{A}	349.97 ± 3.27 ^{A}	367.58 ± 28.34 ^{a}	323.08 ± 17.78 ^{a}	369.56 ± 9.86 ^{a}
- Magnesium	36.31 ± 2.80 ^{B*}	31.87 ± 1.20 ^{B*}	58.87 ± 3.65 ^{A*}	78.45 ± 0.70 ^{b}	56.68 ± 2.74 ^{c}	137.62 ± 2.14 ^{a}
- Iron	0.65 ± 0.07 ^{A*}	0.69 ± 0.01 ^{A*}	0.76 ± 0.08 ^{A*}	1.26 ± 0.04 ^{a}	0.91 ± 0.08 ^{b}	1.01 ± 0.09 ^{b}
- Zinc	0.47 ± 0.10 ^{A}	0.52 ± 0.01 ^{A*}	0.48 ± 0.00 ^{A}	0.52 ± 0.01 ^{a}	0.57 ± 0.01 ^{a}	0.52 ± 0.04 ^{a}

All data were expressed as mean ± standard deviation (SD) of triplicate experiments (n = 3). ND: not detected; TDF: total dietary fiber; SDF: soluble dietary fiber; IDF: insoluble dietary fiber; capital and small letters indicate significant differences ($p < 0.05$) of the same nutrients in young shoots and old leaves, respectively, from different harvesting periods using one-way analysis of variance (ANOVA) and Duncan's multiple comparison test; * indicates significant differences ($p < 0.05$) of the same nutrient between young shoot and old leaves from the same harvesting period using unpaired t-test.
